# BMP-induced non-canonical signaling is upregulated during autophagy-mediated regeneration in inflamed mesothelial cells

**DOI:** 10.1038/s41598-023-37453-x

**Published:** 2023-06-27

**Authors:** Viktória Zsiros, Nikolett Dóczi, Gábor Petővári, Alexandra Pop, Zsófia Erdei, Anna Sebestyén, Anna L. Kiss

**Affiliations:** 1 Department of Anatomy, Histology and Embryology, Semmelweis University Budapest, Tűzoltó u. 58., Budapest, 1094 Hungary; 2 Department of Pathology and Experimental Cancer Research, Semmelweis University Budapest, Üllői út 26., Budapest, 1085 Hungary

**Keywords:** Biochemistry, Cell biology, Molecular biology

## Abstract

Previously, we showed that after Freund’s adjuvant-induced peritonitis, rat mesothelial cells regain their epithelial phenotype through mesenchymal-epithelial transition (MET) accompanied by autophagy. Since bone morphogenetic proteins (BMPs) are well-known MET-inducers, we were interested in the potential expression of BMPs and BMP-induced pathways. Although mesothelial cells expressed lower amounts of BMP7, its level in the peritoneal cavity and mesothelial synthesis of BMP4 were significantly increased during inflammation. BMPR1A and BMPR2 were also significantly expressed. Expression of transforming growth factor beta-activated kinase (TAK1) and c-Jun NH2-terminal kinases (JNK1-JNK2) were more intense than that of phosphorylated Mothers Against Decapentaplegic homolog 1/5 (p-SMAD1/5), confirming that the non-canonical pathway of BMPs prevailed in our model. JNK signaling through B-cell lymphoma-2 (Bcl-2) can contribute to Beclin-1 activation. We demonstrated that TAK1-JNK-Bcl-2 signaling was upregulated simultaneously with the autophagy-mediated regeneration. A further goal of our study was to prove the regenerative role of autophagy after inflammation. We used a specific inhibitor, bafilomycin A1 (BafA1), and found that BafA1 treatment decreased the expression of microtubule-associated protein 1A/1B-light chain 3 (LC3B) and resulted in morphological signs of cell death in inflamed mesothelial cells indicating that if autophagy is arrested, regeneration turns into cell death and consequently, mesothelial cells die.

## Introduction

Intraperitoneal injection of Freund’s adjuvant induces acute peritonitis in rat’s abdominal cavity whereupon mesenteric mesothelial cells losing their epithelial feature undergo significant morphological and molecular changes and differentiate into mesenchymal, macrophage-like cells^1–6^. This biological process is known as epithelial-mesenchymal transition type II (EMT II)^7^, and occurs during inflammatory conditions^8^. After the peak time of inflammation (day 5) mesothelial cells start to regenerate, and by the 11th day, after Freund’s adjuvant injection, the cells regain their original simple squamous phenotype^6^. This process is known as mesenchymal-epithelial transition (MET) which is the reverse process of EMT^9,10^.

EMT and MET can be regulated by several signaling molecules. Members of the transforming growth factor beta (TGF-β) superfamily: inhibins, activins, bone morphogenetic proteins (BMPs), differentiation and growth factors, etc. are well-known modulators of these processes^11^. BMPs are multifunctional growth factors and play important roles in many biological processes^12,13^. Recently, more than 20 types of BMP proteins have been identified and characterized^13^. Their role has been extensively studied in embryonic development as well as in various cellular processes in both postnatal and adult animals^13^. BMP proteins can perform an extremely diverse function which can be explained by several intracellular partners involved in their signaling mechanisms, and by the emerging signaling pathways that can communicate with other pathways providing a wide variety of responses^12^.

To induce signaling as a biologically active ligand, BMP proteins undergo proteolytic cleavage and form homo- and heterodimers. Certain heterodimers, such as BMP2/6, BMP2/7, or BMP4/7, are much more efficient than their homodimers^14^. The active ligands are able to induce signaling through serine/threonine kinase receptors (BMPR-I and -II) with a single transmembrane domain^12^. Several subtypes of receptors are known to mediate BMP signal^14^. From the 3 types of type II receptors, only BMPR2 (BMPR-II) can bind BMP specifically^13,14^. From type I receptors, BMPR1A (BMPR-IA) and BMPR1B (BMPR-IB) are only able to initiate signaling pathways by binding BMP ligand^14^.

BMP can transmit the signal into SMAD-dependent and SMAD-independent pathways^13–15^. Belonging to the canonical route, SMAD factors are the best-known and main intracellular signal transducers of BMP receptors^16^. According to the functional and structural properties of SMADs, three different types of SMAD proteins have been defined: (1) the receptor-regulated SMAD1, -2, -3, -5, and -8 (R-SMAD); (2) a common mediator SMAD4; and (3) the inhibitory SMAD6 and -7^16^. BMPs induce a signal transduction cascade by binding to cell surface receptors and forming a heterotetrameric complex composed of two dimers of type I and type II receptors. The constitutively active type II receptor transphosphorylates the type I receptor allowing the phosphorylation of R-SMADs at serine residues located in their C-terminus^16,17^. R-SMADs involved in BMP signaling are SMAD1, SMAD5, and SMAD8 (SMAD1/5/8). R-SMADs then associate with the co-mediator SMAD4, and this complex translocates to the cell nucleus regulating different target gene expressions^17,18^.

When BMP proteins bind to type I receptors with higher affinity, BMPR1A or 1B helps to recruit BMPR2, forming a heterooligomer complex, which ultimately stimulates SMAD-independent signaling pathway^14^. The so-called X-linked inhibitor of apoptosis protein (XIAP) that is involved in inhibiting apoptosis, acts as an adapter protein between type I receptor and TGF-β activated binding proteins (TAB), that later activates TGF-β activated tyrosine kinase 1 (TAK1). TAK1 was first identified as a mitogen-activated protein kinase kinase kinase (MAP3K). TAK1 is known to modulate inflammation and cell viability via activating downstream effectors such as NF-*κ*B and mitogen-activated protein kinases (MAPKs)^19^. This protein then triggers various SMAD-independent pathways such as NF-κB or MAP kinase e.g., JNK signaling mechanisms^14,20^. MAP kinases are highly conservative serine/threonine protein kinases having crucial role in the signal transduction system^21^. The c-Jun NH2-terminal kinases (JNKs) are members of six sub-families of MAPKs identified in mammalian cells. JNKs were initially identified as stress-activated protein kinases (SAPKs) in the mouse liver treated with cycloheximide to induce inflammation and apoptosis, but later they were renamed to emphasize their relationship with c-Jun^21^. There are three genes that encode JNKs in mammals: the widely expressed *Jnk1*, *Jnk2* and *Jnk3*, which is only expressed in the brain, testis, and heart^21^. The relationship between BMP-linked non-canonical pathways and autophagy is a poorly studied field. Only limited information is available, but it is known that BMP ligands can promote autophagy through non-canonical, SMAD-independent pathways^22–24^. There are studies indicating that JNK pathway plays an important role in various forms of autophagy^21,25^. In our present experimental work, we also found that the non-canonical TAK1-JNK-Bcl-2 signaling pathway induced by BMPs was activated parallel to the intense autophagy during the recovery of inflamed mesothelial cells.

TGF-β is known to be an inducer of EMT, while BMP proteins most likely induce MET^15,26–28^. Our question was whether BMP proteins play any role in the regeneration of mesothelial cells (MET). To find out whether BMPs are produced by mesothelial cells and whether these cells express BMP ligand-specific receptors, we applied immunocytochemistry (IC), Western blot (WB), and statistical analyses. Even though our results showed that the major MET inducer, BMP7, was not considerably expressed in mesothelial cells, we could detect it in the peritoneal fluid, and its amount was gradually increased after the Freund’s adjuvant injection. However, mesothelial cells expressed BMP4 in a significant amount during the days of inflammation which could initiate different signaling events in the presence of BMP7, BMPR1A and BMPR2 in the cells. BMP2 and BMP4 preferentially bind to BMPR1A and BMPR1B and recruit type II receptors^16,17^ which interaction stimulates the non-canonical signaling pathways of BMPs^14^. Our results clearly prove that the non-canonical pathway induced by BMP factors was much more intense than the canonical pathway represented by p-SMAD1/5 in our model system. TAK1 and JNK1-JNK2 kinases showed strong expression at the 3rd day of inflammation and in the days of the regeneration. The anti-apoptotic Bcl-2 was also expressed in mesothelial cells, its amount increased with the beginning of the recovery. It is known that after the phosphorylation of Bcl-2, it can release the autophagic protein Beclin-1, which is an important factor for the induction of autophagy^21^. In correlation with the literary data and with our previous work, we found that the TAK1-JNK-Bcl-2 signaling pathway was upregulated during the cytoprotective autophagy-directed regeneration in the inflamed mesothelial cells.

Autophagy was previously thought to be one form of programmed cell death and was also referred to as type 2 cell death. However, it appeared recently that autophagy is rather a degradative process^29^. It is a highly conserved, cellular recycling process that occurs in all eukaryotic cells, and plays a very important role in many biological processes, like cell survival and controlling basic vital functions of cells^30^. Our previous results show, that after the peak time of Freund’s adjuvant-induced inflammation, with the start of regeneration the autophagic activity of mesothelial cells is highly elevated^6^. In our previous paper, we proved that in the early stage of inflammation (day 3), the expression of Beclin-1 is significantly increased and then gradually decreased from day 8, indicating that one of the essential factors for the induction of autophagy is present and freed from inhibition in the mesothelial cells. Furthermore, we investigated the expression level of microtubule-associated protein 1A/1B-light chain 3 (LC3B) as one of the most important membrane markers of autophagic organelles. Our previous WB data clearly showed that the amount of the membrane-bound and cytosolic LC3B change inversely: as the autophagic degradation is accelerated in the mesothelial cells the level of the cytosolic LC3B-I increases, while the membrane-bound LC3B-II entirely disappears indicating that the membrane bound LC3B-II is also degraded by progressive autophagy^6^. To verify that autophagy indeed plays a crucial role in restoring the original morphology of squamous mesothelial cell, we used bafilomycin A1 (BafA1) treatment and followed the regenerative process. BafA1, the most commonly applied inhibitor of autophagy, prevents the maturation of autophagic vacuoles by inhibiting the fusion between autophagosomes (APs) and lysosomes^31^. Being a specific inhibitor of V-ATPase in cells, it inhibits the acidification of endosomes and lysosomes^31^. To inhibit autophagy in our in vivo system, BafA1 was injected into the rats’ peritoneal cavity. Our results prove that BafA1 successfully blocked the maturation of APs and decreased the expression level of LC3B. Furthermore, we could detect dramatic morphological changes in the inflamed mesothelial cells, derived from BafA1 treated rats, showing the typical signs of cell death, which was caspase-independent in our model. These results confirmed that mesothelial cells could not recover from inflammation after BafA1 treatment; instead, they died supporting the indispensable role of autophagy in regeneration.

## Materials and methods

### Animal models and ethics statement

60- to 70-day-old (200–300 g) male Sprague–Dawley rats (Charles River Research Models and Services, Sulzfeld, Baden-Wurttemberg, Germany) were used for each in vivo experiment (n = 5 in each group; experiments were repeated 3 times) following the progress of inflammation after Freund’s adjuvant injections and were maintained under specific pathogen-free conditions (air humidity of 45% and 21 °C). Each animal experiment was carried out in accordance with the Guide for the use of Adjuvants in Research of the National Institutes of Health and approved by the Institutional Animal Care and Use Committee of the University of Massachusetts Amherst (ARAC Guidelines 2010) and the Institutional Animal Care and Use Committee of Semmelweis University. All methods reported here comply with ARRIVE guidelines. All efforts have been made to minimize suffering (SEMÁB-B/098/2015; PE/EA/00877-6/2022).

The animals were anesthetized with a xylazine-ketamine mixture (intramuscular injection; (0.6 mg/250 g animals: 0.2 mg xylazine (20 mg/ml) + 0.4 mg ketamine (50 mg/ml)), and then decapitated. Mesenteries were isolated from the abdominal cavity of the animals at various time points followed Freund’s adjuvant treatments (3, 5, 8, 11 days; D: day; D3, D5, D8, D11), and were used for further experiments. Mesenteries isolated from untreated animals were used as control samples (CTRs).

### Materials

Freund’s adjuvant: To induce acute peritonitis, 1 ml complete Freund's adjuvant (Sigma-Aldrich^®^, Saint Louis, Missouri, USA) was injected into the peritoneal cavity of the animals.

BafA1: For inhibiting autophagy, BafA1 (Santa Cruz Biotechnology, Inc., Dallas, Texas, USA) was used according to the information of the manufacturer and the literature: 0.25 mg BafA1 was dissolved in 2.5 ml of DMSO (dimethyl sulfoxide)^32,33^, then this solution was further dissolved in 2.5 ml of distilled water. This BafA1-containing mixture (at a dose of 1 mg/kg per animals)^32,33^ was injected intraperitoneally into the animals in 1, 2 and 2.5 ml doses depending on the experimental group. The in vivo effect of BafA1 was followed in control (CTR) and inflamed (D3, D5, D8) animals. Control animals were treated with BafA1 for 2 and 4 h (CTR_1B_, CTR_2B_). Freund’s adjuvant injected rats were treated for 4 h on the 3rd day of inflammation (D3_B_), another group was injected with BafA1 on the 4th day of inflammation and observed after 24 h on the 5th day (D5_B_). In the last experimental group, 8 days after injection of Freund's adjuvant (D8), several treatments were used. In the first group (D8_1B_) the animals were injected with 2.5 ml of BafA1 cocktail only on day 4 of inflammation. Then we combined the treatments, and since the animals were vaccinated several times during the inflammatory period, we reduced the amount of injected BafA1. Two rats were treated daily with 1 ml of BafA1 mixture from day 4 of inflammation until day 7 (D8_2B_). Two animals were injected with 1.5 ml BafA1 on day 5 and 6 (D8_3B_); and further two other rats were injected on day 6 and 7 (D8_4B_). The scheme of these treatments is summarized in the table below (Table1).

**Table 1:** Summary of BafA1 treatments.

Groups	Freund’s adjuvant injection	Number of BafA1 injections	Days of BafA1 injection	Duration of BafA1’s effect
CTR_1B_	−	1x	Day 0	2 h
CTR_2B_	−	1x	Day 0	4 h
D3_B_	+	1x	Day 3	4 h
D5_B_	+	1x	Day 4	24 h
D8_1B_	+	1x	Day 4	96 h
D8_2B_	+	4x	Day 4–day 7	96 h
D8_3B_	+	2x	Day 5 and 6	72 h
D8_4B_	+	2x	Day 6 and 7	48 h

Details about the number, days, and duration of BafA1 treatments.

### Antibodies for immunocytochemistry and Western Blot analysis

IC and WB analyses were used to detect proteins which are indirectly or directly involved in the BMP signaling pathways and typical for apoptosis. The followings antibodies were applied: rabbit anti-BMP4 polyclonal (IC: 1:200; Abcam plc, Cambridge, UK), rabbit polyclonal anti-BMP7 (IC: 1:200, WB: 1:100; Abcam plc) (49 kDa), rabbit polyclonal antibody to BMPR1A (IC: 1:200, WB: 1:200; Abcam plc) (58 kDa), mouse monoclonal to BMPR2 (IC: 1:100, WB: 1:500; Abcam plc) (115 kDa), rabbit polyclonal anti-p-SMAD1/5 (Ser463/465) (IC: 1:150; Cell Signaling Technology^®^, Danvers, USA), rabbit polyclonal to TAK1 (IC: 1:150; GeneTex, Inc., Irvine, US), rabbit polyclonal anti-JNK1-JNK2 (IC: 1:150; GeneTex, Inc.), rabbit polyclonal to Bcl-2 (IC: 1:150; GeneTex, Inc.), rabbit polyclonal anti-LC3B (IC: 1:200; Cell Signaling Technology^®^), rabbit polyclonal cleaved caspase-3 (Asp175) antibody (IC: 1:200; Cell Signaling Technology^®^), and HRP-conjugated and unconjugated mouse monoclonal anti-beta-actin (WB: 1:10,000; Abcam plc) (42 kDa) as primary antibodies. In our immunocytochemical studies (confocal microscopy) species-specific Alexa dyes (anti-rabbit and anti-mouse IgG Alexa Fluor 488, 1:200; Molecular Probes^®^, Leiden, The Netherlands) were used to visualize the corresponding antibodies. For WB analyses species-specific peroxidase-conjugated anti-rabbit and anti-mouse secondary antibodies (Amersham, GE Healthcare Biosciences, Pittsburgh, USA) were applied in a dilution of 1:5000 and 1:10,000, respectively.

### Morphological examinations

#### Fixation and preparation of the mesentery for light and electron microscopy

Mesenteries were fixed with a 1:1 mixture of 2% glutaraldehyde and 2% osmium tetroxide in 0.1 M cacodylate buffer at 4 °C, were washed with 0.1 M cacodylate buffer, then the surrounding adipose tissue was removed. After washing, the samples were dehydrated in an ascending ethanol series and embedded in araldite following the methodology based on our previously described protocol^1,6,34^. For light microscopy, semithin sections were cut from the embedded samples on a Reichert Ultracut ultra-microtome, stained with toluidine blue, and examined in a conventional light microscope (Carl Zeiss), photographed with a Zeiss Axiocam HRc camera, and analyzed with Axiovision. For electron microscopy, ultrathin sections were prepared on Ultracut ultramicrotome and contrast-stained with uranyl acetate and lead (II) nitrate. Afterwards, the samples were photographed using a Hitachi H-7600 and a JEOL JEM-1200EX II. transmission electron microscope and examined by iTEM analysis program. Each light and electron microscopical image for publication has a resolution of 300 pixels/inch, and only brightness and contrast were adjusted on the entirety of them by Adobe Photoshop 7.0.1. Images used for evaluation were not modified. Supporting the effect of BafA1 treatment on the regeneration of mesothelial cells, statistical analysis was used on the light microscopical photographs to compare the number of living versus dead cells in different groups for a unified area. Cells were considered to be alive if they had an intact plasma membrane edge, no cytoplasm fragments had broken off, and the cell nucleus was not condensed or loosened completely.

### Confocal microscopy

#### Sample preparation for confocal immunocytochemical studies

Isolated mesenteries were fixed in 4% paraformaldehyde (PFA) in 0.2 M phosphate buffer (PBS), pH: 7.4 at 24 °C for 1 h and were stored in 1% PFA in the same buffer at 4 °C until further processing. For immunolabeling on semi-thin frozen sections, we applied a modified Tokuyashu technique^35^. The PFA-fixed samples were washed with 0.05 M glycine in PBS. After removing the adipose tissue, they were embedded in 10% gelatin (30 min, 37 °C). Then small blocks (3 * 3 mm) were formed. For cryoprotection, the blocks were incubated in 2.3 M sucrose (overnight, 4 °C), subsequently mounted onto aluminum pins and frozen in liquid nitrogen following the methodology used in our previous studies^1,6,34^. The 0.6 μm thick frozen sections were cut with Leica Ultracut S ultramicrotome (Vienna, Austria).

#### The process and evaluation of fluorescent immunolabeling

The semi-thin sections were washed with PBS (3 times, 10 min) and glycine-PBS, then blocked with 1% bovine serum albumin (BSA) in phosphate buffer for 15 min and incubated with the primary antibodies overnight (4 °C) following the habitual steps describing in our previous works^6,34^. To visualize the primary antibodies, species-specific Alexa Fluor conjugated antibodies (Alexa Fluor 488, 1 h, dark) were used. Nuclei were stained by Vectashield DAPI (Vector Laboratories Inc. Burlingame, California, USA). The sections were examined by a laser scanning (confocal) microscope Zeiss LSM-780 (Carl Zeiss Technika Kft., Budaörs, Hungary) and analyzed by the ZEN program. Then images for publication were processed with Adobe Photoshop 7.0.1. software for brightness and contrast adjustments, which were applied to the entirety of images, but images used for evaluation were not modified. Each fluorescent image has a resolution of 300 pixels/inch. Among the used markers, those whose comparison was relevant were subjected to statistical analyses to support the obtained results. Our aim was to quantify and compare the signal intensities detected by IC. Cells (intact plasma membrane boundaries, visible nucleus) were encircled manually for each group (CTR-D11) and the intensity of the signal in green channel was measured using Fiji^36^ program. The intensity results were grouped according to the duration of inflammation and regeneration.

### Biochemical experiments

#### Isolation of mesothelial cell lysate

For biochemical analyses, isolated mesenteries were further processed according to Hjelle et al.^37^. Control and Freund’s adjuvant injected peritoneal cavities were washed with PBS (for collecting peritoneal fluid), then isolated mesentery was incubated in 0.2% collagenase type II (Sigma-Aldrich^®^, Saint Louis, Missouri, USA) in DMEM/F12 medium for 60 min at 37 °C. After collagenase digestion, the samples were washed interposing centrifugations (1000 rpm, 3 × 10 min, 4 °C), and stored at − 80 °C until further processing for biochemical investigation. The isolated mesothelial cell lysates were dissolved in lysis buffer containing 50 mM TRIS–HCl (pH: 7.5), 150 mM NaCl, 2 mM EDTA, 200 mM Na_3_VO_4_, 1 mM NaF, 1% Nonidet P-40 and protease inhibitor mixture (Complete Mini, Roche, Mannheim, Germany), kept for 60 min on ice, then centrifuged (12,000 rpm, 20 min, 4 °C) to remove insoluble material. The protein content of the supernatant (mesothelial cell lysate) was determined by the BCA method^38^, and the samples were diluted to 2 mg/ml.

#### Western blot analysis

After adding tris-SDS buffer (0.5 M TRIS pH: 6.8, 10% glycerol, 2% SDS, 0.00125% bromophenol blue, 0.5% mercaptoethanol) to the mesothelial cell lysates (in 1:1 ratio), the lysates were boiled for 4 min. Subsequently, the proteins were separated by electrophoresis (10% polyacrylamide gel, 200 V, 30–40 min). To transfer the proteins to the nitrocellulose membrane, the gel and membrane were blotted at 100 V for 1 h under continuous cooling in blot buffer. The membranes were washed in 0.0005% Tween-PBS buffer, then were stained with Ponceau S (Sigma-Aldrich^®^, Saint Louis, Missouri, USA) for instant visualization of the protein fractions, and blocked with 5% non-fat dry milk, washed, and incubated with the primary antibodies (overnight, 4 °C) following the usual methodology we described previously^6,34,39,40^. β-actin were used as an internal loading control for lysates. The membranes were incubated with specific peroxidase-conjugated secondary antibodies (Amersham, GE Healthcare Biosciences, Pittsburgh, USA) for 2 h to visualize the primary antibodies. For detecting the labeled proteins, the membranes were treated with a chemiluminescent solution (Luminata Forte Western HRP Substrate, Millipore, USA), and the immune response was detected using X-ray films, LI-COR C-Digit Blot scanner and BioRad ChemiDoc Imaging System. The relative densities for the evaluation of the obtained WB data were determined from the results of three independent measurements using the ImageJ software (area, percent) (U.S. National Institutional of Health, Bethesda, Maryland) and analyzed by Microsoft Excel 2013 program. The samples derived from the same experiments and the gels/blots were processed in parallel. In each densitometry, relative density of the observed proteins was compared to the control. The density results were grouped according to the duration of inflammation and regeneration.

### Statistical analysis

The data were tested with Kolmogorov–Smirnov test, and in all cases, we had to reject the null hypothesis of normal distribution of the data set. Therefore, the non-parametric Kruskal–Wallis test was used to examine whether the data of experimental groups come from the same distribution. In cases where the null hypothesis was rejected, post-hoc pairwise comparison was used to find the significantly different data sets (*p < 0.05; **p < 0.01, ***p < 0.001). Means and standard deviations are visible on the diagrams. Tables with the exact cell numbers, means, and standard deviations can be found in Supplementary Fig. 1. All statistical analyses were performed with SPSS program.

## Results

### Changes in the expression level of BMP factors and receptors after Freund’s adjuvant injection

Among the members of TGF-β superfamily, as key regulators of EMT and MET, BMP proteins are mainly responsible for MET^15,26–28^. Therefore, we studied whether BMP factors are produced by mesothelial cells, and whether they express any of BMP ligand-specific and signal transducing receptors using protein expression analyses of BMP4, BMP7, BMPR1A and BMPR2.

#### Expression of BMP4 and BMP7 in mesothelial cells

**Figure 1:**
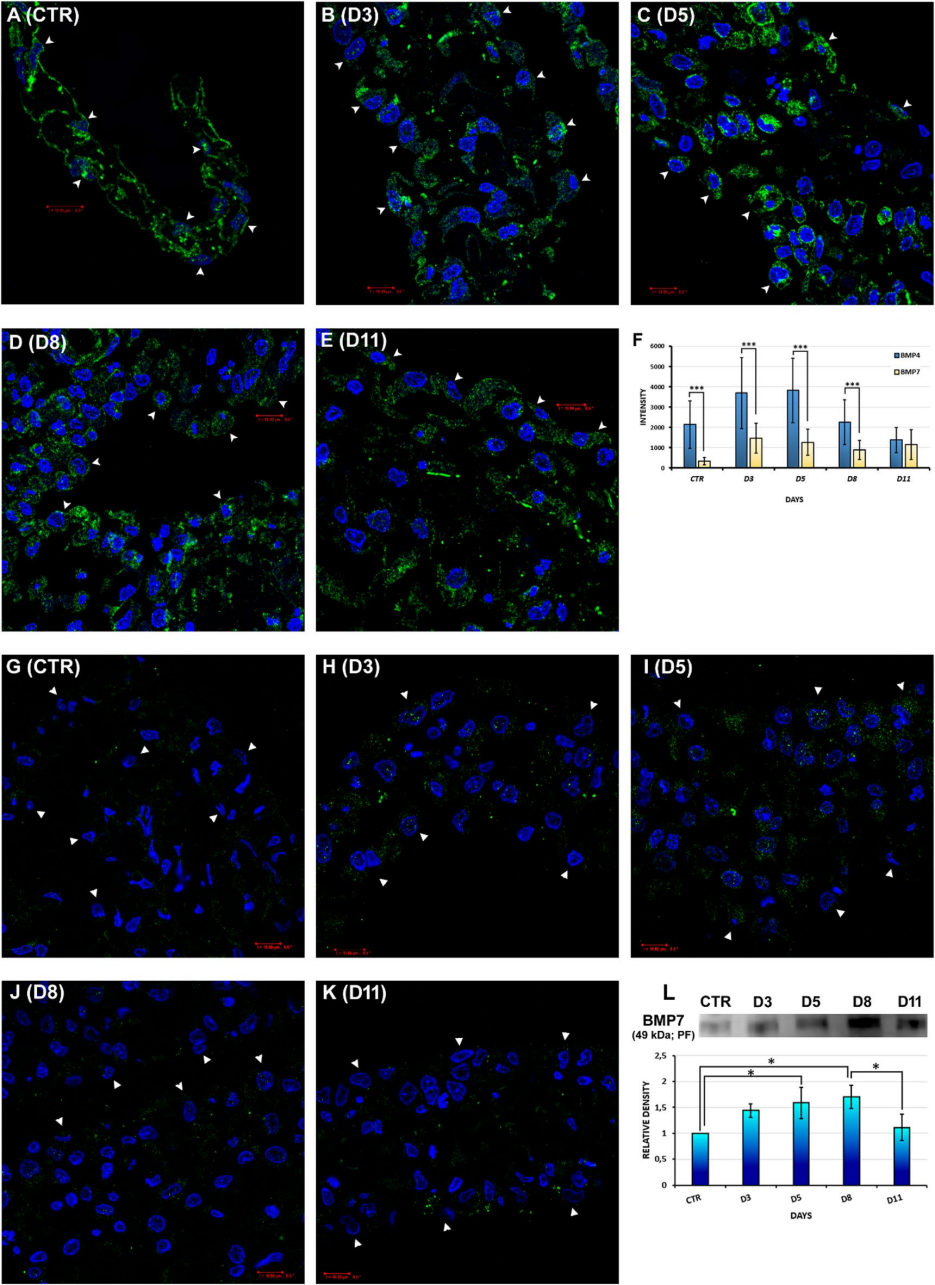
BMP4 and BMP7 production of mesothelial cells (IC, WB, and statistical analyses). (**A**) BMP4 immunopositivity was already found in healthy, control mesothelial cells (CTR). (**B**,**C**) On day 3 (D3), the BMP4 expression increased and showed a strong signal on day 5 (D5). (**D**,**E**) At the 8th and 11th day after Freund’s adjuvant injection (D8, D11), BMP4 was still expressed in regenerating mesothelial cells and in the connective tissue cells as well. (**F**) Statistical comparison of BMP4 and BMP7 expression in mesothelial cells (***p < 0.001). (**G**) Control mesothelial cells expressed small amount of BMP7 (CTR). (**H**,**I**) On the 3rd and 5th days of inflammation (D3, D5), BMP7 expression increased both in the mesothelial and connective tissue cells. (**J**,**K**) On day 8 and 11 (D8, D11), BMP7 positivity was slightly decreased. (**L**) BMP7 was remarkably presented in the peritoneal fluid, its expression level increased after Freund's adjuvant injection (see densitometry below). The statistical analysis shows the densitometry analyzed WB results of BMP7 in peritoneal fluid samples (*p < 0.05) (arrowheads: mesothelial cells; PF: peritoneal fluid; blue: DAPI; green: (**A**–**E**) BMP4, (**G**–**K**) BMP7; WB image was cropped; original blot is presented in Supplementary Fig. S1; data of the statistical analyses and cell numbers of the groups are presented in Supplementary Tables S1, S2). Bar ~ 10.80 µm.

BMP4 immunopositivity could be found already in healthy mesothelial cells with confocal microscope (Fig. 1A,F). By the time of inflammation (D3, D5), BMP4 expression increased (Fig. 1B,C,F), and gradually decreased as the regeneration started (D8, D11) (Fig. 1D–F).

Our immunocytochemical results revealed that control mesothelial cells expressed small amount of BMP7 (Fig. 1G,F), while on the 3rd day of inflammation stronger, dot-like BMP7 signals could be detected in the mesentery (Fig. 1H,F). The expression of BMP7 on day 5 was similar to that observed on day 3 (Fig. 1I,F), but this expression was significantly lower (D3, ***p = 8.22 * 10^–7^; D5, ***p = 7.91 * 10^–6^) then the detected staining intensity of BMP4 on these days (Fig. 1F). BMP7 showed discrete, smaller expression at the 8th day in mesothelial cells (Fig. 1J,F), but the expression intensity of BMP7 increased again at the end of the regeneration (D11) (Fig. 1K,F). Our statistical analysis revealed that there were significant differences between the expression intensity of BMP4 and BMP7 not only in control cells (CTR, ***p = 8.02 * 10^–18^), but also in different days of inflammation (D3-D8; D8, ***p = 1.82 * 10^–9^), except for the 11th day of regeneration (D11) (Fig. 1F). However, the intracellular BMP7 protein level was lower (undetectable by WB in mesothelial cell lysate), the results from the peritoneal fluid underlined the importance of potential BMP7 secretion. Our WB analyses clearly showed that BMP7 was expressed even in the control animals’ peritoneal fluid, and its level gradually increased after the Freund’s adjuvant injection (D3, D5) (Fig. 1L). Its expression reached the maximum on the 8th day when the regeneration of mesothelial cells started (Fig. 1L). The statistical analysis of the relative densities from WB results proved that BMP7 densities measured on days 5 and 8 showed a significant difference with the control (CTR-D5, *p = 0.041357; CTR-D8, *p = 0.014116), and the difference between days 8 and 11 was also significant (D8-D11, *p = 0.038283) (Fig. 1L).

#### Expression of BMP-specific receptors in mesothelial cells

**Figure 2:**
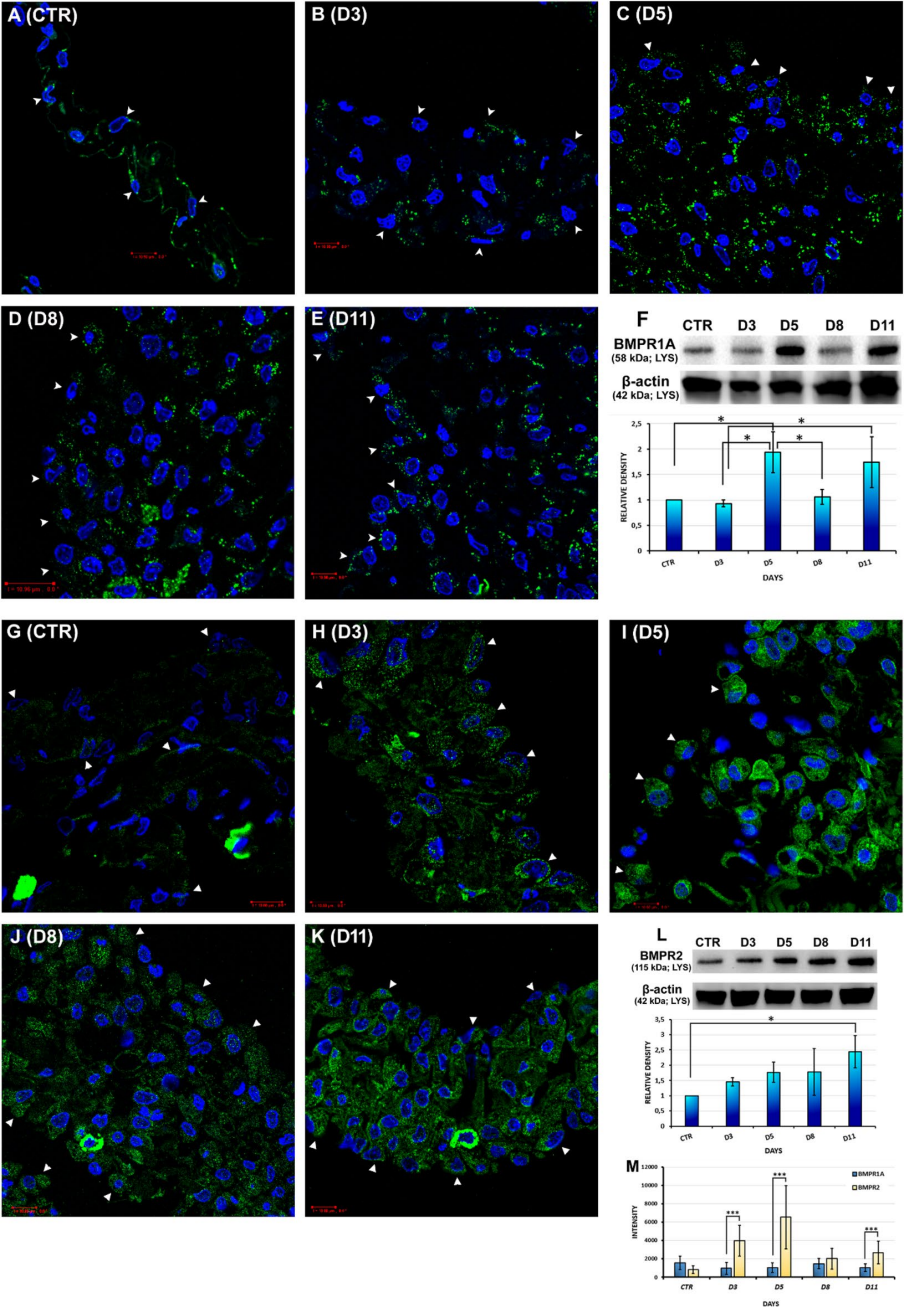
Expression of BMPR1A and BMPR2 was increased in inflamed and recovering mesothelial cells (IC, WB, and statistical analyses). (**A**) Dot-like BMPR1A expression could be detected in control mesothelial cells (CTR). (**B**) On the 3rd day after Freund’s adjuvant injection (D3), BMPR1A expression was weakly decreased. (**C**) On the 5th day (D5), the presence of BMPR1A was more pronounced in the mesentery, while just a moderately increased in mesothelial cells. (**D**) On day 8 (D8), the expression of BMPR1A was slightly stronger. (**E**) At the end of the regeneration (D11), mesothelial cells were still BMPR1A positive. (**F**) The WB analysis showed that BMPR1A was already expressed in control mesothelial cells (CTR), its expression level was high on day 5 and 11 (D5, D11). Statistical analysis of WB densitometry data on BMPR1A expression in mesothelial cell lysates (*p < 0.05). β-actin was used as a loading control. (**G**) BMPR2 expression was barely detectable in control cells (CTR). (**H**) On day 3 (D3), strong, specific, punctate-like BMPR2 signals could be observed in the cytoplasm of mesothelial cells. (**I**) On the 5th day (D5), the receptor expression was enhanced. (**J**,**K)** As the regeneration started (D8, D11), BMPR2 expression was still intense. (**L**) WB results and the densitometry showed that the BMPR2 expression level was gradually increased over time. Statistical analysis of WB densitometry data on BMPR2 expression in mesothelial cell lysates (*p < 0.05). β-actin was used as a loading control. (**M**) Statistical comparison of BMPR1A and BMPR2 expression in mesothelial cells (***p < 0.001). (Arrowheads: mesothelial cells; LYS: mesothelial cell lysate; blue: DAPI; green: (**A**–**E**) BMPR1A, (**G**–**K**) BMPR2; WB images were cropped; original blots are presented in Supplementary Fig. S2; data of the statistical analyses and cell numbers of the groups are presented in Supplementary Tables S1, S2). Bar ~ 10.80 µm.

The immunocytochemical results showed punctate-like BMPR1A expression in control mesothelial cells (Fig. 2A). The changes in the receptor expression changes were confirmed by WBs and their statistical analyses (Fig. 2F,M). BMPR1A expression slightly decreased at the 3rd day after Freund’s adjuvant treatment (Fig. 2B,F,M). On day 5, significantly increased expression of BMPR1A was detected with WBs and their statistical analyses (Fig. 2F,M) compared to IC analyses, where mesothelial cells showed definitive, punctate-like BMPR1A signals in the mesothelial cells (Fig. 2C).

On the 8th day, BMPR1A expression was found to be a little stronger with IC as on day 5 (Fig. 2D). WB results, however, showed a slight decrease (Fig. 2F). At the end of the regeneration (D11), BMPR1A expression could be still detected in the mesothelial cells (Fig. 2E,F,M). The statistical analysis showed significant differences in the BMPR1A relative density between the control sample and day 5 (*p = 0.019482), day 3 and day 5 (*p = 0.012782), day 3 and day 11 (*p = 0.04521) and day 5 and 8 (*p = 0.02958) (Fig. 2F).

Regarding BMPR2 expression analyses, we found that the receptor was barely present, but expressed in control, untreated mesothelial cells (Fig. 2G,L,M). After 3 days of Freund's adjuvant treatment, BMPR2 expression increased (Fig. 2L,M), and specific, punctate-like signals could be detected in the rounded mesothelial cells (Fig. 2H). On day 5, the expression of BMPR2 was further enhanced (Fig. 2L,M) and a very intense signal could be seen in the mesentery even in the connective tissue cells as well (Fig. 2I). On the 8th day, IC showed a weaker signal intensity when compared to that obtained on day 5 (Fig. 2J,M), but in the WB analysis a slight increase was found (Fig. 2L). We could still detect an intense BMPR2 immunolabelling (Fig. 2K,M), as well as a high BMPR2 expression level in the mesothelial cell lysate on the 11th day (Fig. 2L). The statistical analyses of BMPR2 WB densitometry results showed significant difference between control and day 11 samples (*p = 0.01755) (Fig. 2L). Our statistical analysis comparing the signal intensities of BMPR1A and BMPR2 revealed that there were significant differences not only at the 3rd day of inflammation (D3, ***p = 1.06 * 10^–13^) but also at day 5 (D5, ***p = 3.51 * 10^–15^) and day 11 (D11, ***p = 1.45 * 10^–6^), BMPR2 expression was significantly intense (Fig. 2M).

### Detection of BMP-induced canonical and non-canonical signaling pathways in mesothelial cells during EMT-MET

It is known that BMP can stimulate both SMAD-dependent and SMAD-independent pathways^13–15^. The high-affinity BMP protein binding to type I receptors facilitates BMPR2 recruitment, which consequently stimulates SMAD-independent, non-canonical signaling^14^. Therefore, we were curious as to which pathway is activated by BMP4 and BMP7 in our model system. To answer this question, we performed immunocytochemical and statistical analyzes with the participating factors of the canonical and non-canonical pathway, including p-SMAD1/5 (Ser463/465), TAK1, JNK1-JNK2 and Bcl-2.

#### Expression changes of p-SMAD1/5 and TAK1 during inflammation and regeneration in mesothelial cells

**Figure 3:**
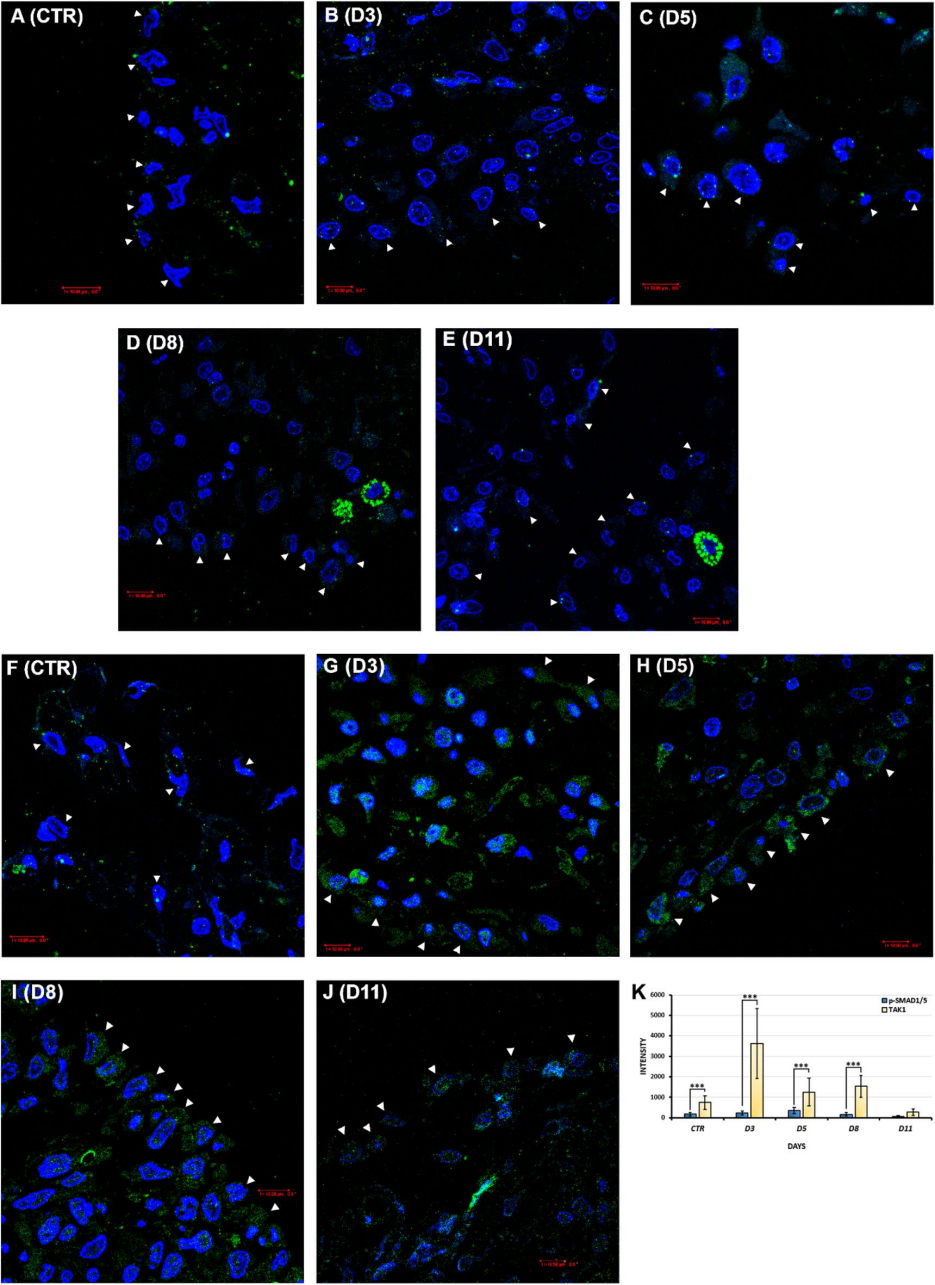
Predominance of BMP-induced SMAD-independent over SMAD-dependent signaling pathway in mesothelial cells (IC and statistical analysis). (**A**) A few, dot-like signals indicating p-SMAD1/5 could be observed in the cytoplasm of control mesothelial cells (CTR). (**B**,**C**) The expression of p-SMAD1/5 was not considerably enhanced on day 3 and 5 (D3, D5), some definite dots could be detected in the cytoplasm and around the nuclei. (**D**,**E**) On the 8th and 11th day (D8, D11), p-SMAD1/5 expression intensity decreased, only the mast cells glowed with their autofluorescence in the connective tissue. (**F**) TAK1 expression could be detected in control, healthy mesothelial cells (CTR). (**G**–**I**) Strong TAK1 expression was seen on days 3, 5 and 8 of inflammation (D3-D8). Sometimes this TAK1 expression correlating green, fluorescent signal even suppressed the blue color of the cell nuclear DAPI staining. (**J**) On the 11th day (D11), the expression of TAK1 decreased, the mesothelial cells showed only a weak TAK1 immunopositivity. (**K**) Statistical comparison of p-SMAD1/5 and TAK1 signal intensities in mesothelial cells (***p < 0.001). (Arrowheads: mesothelial cells; blue: DAPI; green: (**A**–**E**) p-SMAD1/5, (**F**–**J**) TAK1; data of the statistical analyses and cell numbers of the groups are presented in Supplementary Table S1). Bar ~ 10.80 µm.

In the control mesothelial cells, the expression of p-SMAD1/5 (Ser463/465) was low, a few, punctate signals could be detected in their cytoplasm (Fig. 3A,K). The level of p-SMAD1/5 did not increase dramatically during 3 and 5 days of inflammation either (Fig. 3K). Some definite dots could be identified in the cytoplasm and in or around the nucleus of the cells (Fig. 3B,C). On the 8th day after Freund's adjuvant injection, the expression of p-SMAD1/5 was remained very low, most of the definitive, dot-like signals disappeared from the mesothelial cells (Fig. 3D,K). At the end of the regeneration (D11), p-SMAD1/5 expression was barely detectable (Fig. 3E,K). By studying the expression of TAK1, we found that the control mesothelial cells were TAK1 immunopositive. Definitive, punctate-like dots were visible in their thin cytoplasm (Fig. 3F,K). The expression of TAK1 clearly increased during the days of inflammation following Freund's adjuvant treatment compared to the control (Fig. 3K). During the 3rd, 5th, and 8th days of inflammation, TAK1 showed a strong signal intensity and was diffusely stained in the cytoplasm of almost all cells of the mesentery. Sometimes the green channel showing TAK1 expression related staining intensity was even visible through the DAPI-labeled cell nuclei (Fig. 3G–I). However, on the 11th day, the expression of TAK1 decreased, and again only dot-like immune signals could be detected in the mesothelial cells (Fig. 3J,K). Our statistical analysis showed that there were significant differences between the signal intensities of p-SMAD1/5 representing the canonical pathway and TAK1 representing the SMAD-independent pathway in almost every group: in the control samples (CTR, ***p = 3.81 * 10^–5^), on the 3rd (D3, ***p = 3.47 * 10^–21^), 5th (D5, ***p = 4.89 * 10^–8^) and 8th days of inflammation (D8, ***p = 1.95 * 10^–8^) (Fig. 3K).

#### Monitoring the expression of JNK1-JNK2 and Bcl-2 factors on different days of inflammation and regeneration

**Figure 4:**
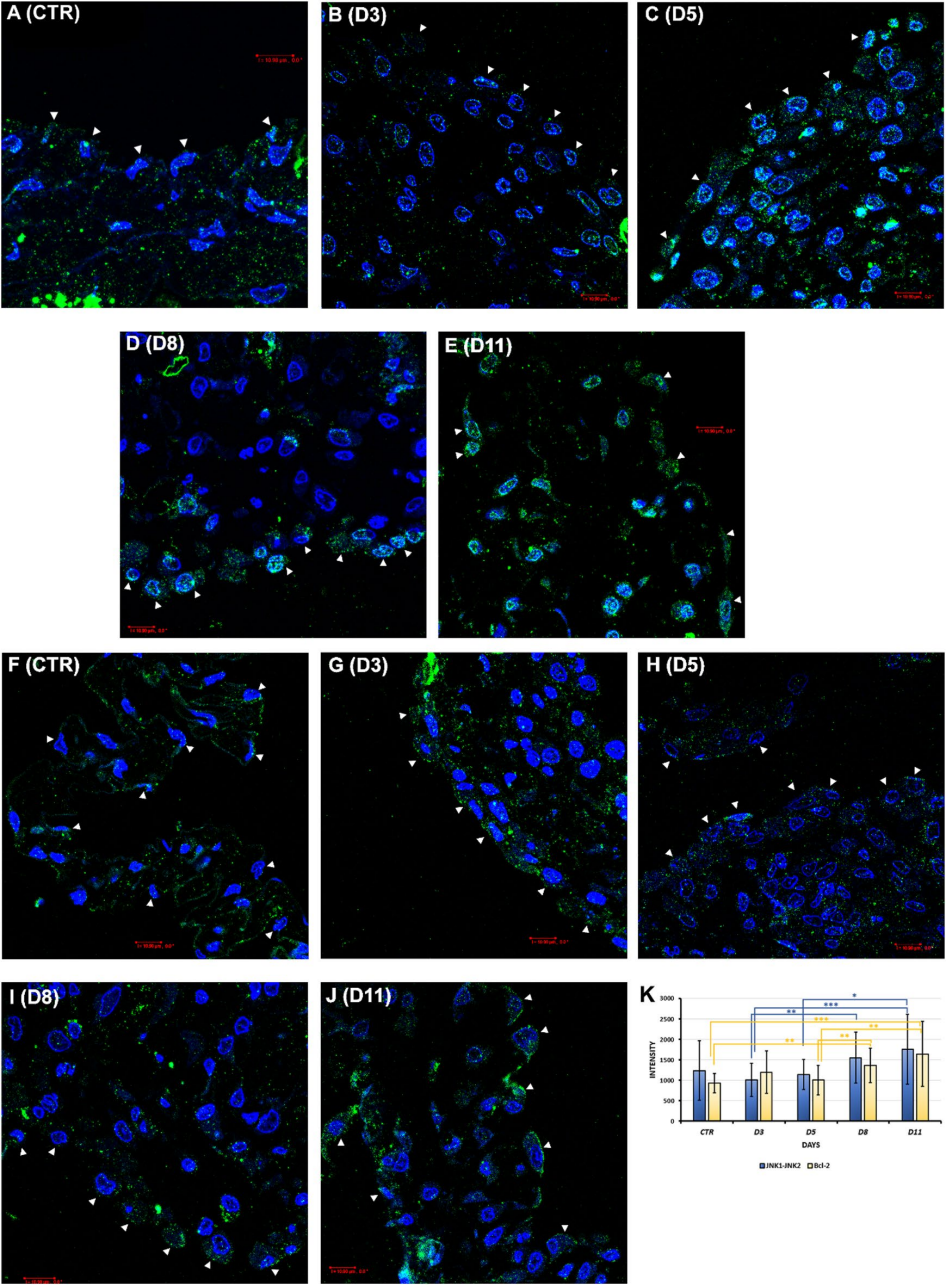
Examination of the expression of JNK1-JNK2 and Bcl-2 in relation to the non-canonical signal of BMPs during EMT-MET (IC). (**A**) JNK1-JNK2 expression could be detected in the untreated mesothelial cells (CTR). (**B**) The expression intensity did not change much on the 3rd day (D3), nice, punctate-like immunopositivity could be seen in the cells. (**C**–**E**) From the 5th day after Freund's adjuvant injection (D5, D8, D11), the expression of JNK1-JNK2 kinases was increased and remained intense with the end of the regeneration. The signal intensity of JNK1-JNK2 was so strong on these days that they were visible at the level of the cell nucleus. (**F**) Bcl-2 immunopositive cells could be observed in the mesothelial cells of the control mesentery (CTR). (**G**) The expression of Bcl-2 was increased on the 3rd day after Freund's adjuvant treatment (D3). (**H**) On the 5th day (D5), Bcl-2 signal intensity was slightly decreased, but it still showed definitive immunopositivity in the cytoplasm of the mesothelial cells. (**I**,**J**) With the beginning of the recovery, Bcl-2 was strongly expressed on day 8 and 11 (D8, D11). (**K**) Statistical analysis of JNK1-JNK2 and Bcl-2 signal intensities in mesothelial cells (*p < 0.05; **p < 0.01, ***p < 0.001). (The blue square brackets show the significant differences in the intensities of JNK1-JNK2, the yellow square brackets show the significant differences in the intensities of Bcl-2). (Arrowheads: mesothelial cells; blue: DAPI; green: (**A**–**E**) JNK1-JNK2, (**F**–**J**) Bcl-2; data of the statistical analyses and cell numbers of the groups are presented in Supplementary Table S4). Bar ~ 10.80 µm.

The mesothelial cells of the untreated, control mesentery expressed JNK1-JNK2 kinases (Fig. 4A,K). This expression slightly decreased on the 3rd day of inflammation (Fig. 4K), but we could detect nice, dot-like signals in the cytoplasm of the cells (Fig. 4B). From the 5th day of Freund's adjuvant injection, the expression level of JNK1-JNK2 gradually increased and even strengthened until the 11th day of regeneration (Fig. 4C–E,K). The increased expression of JNK kinases was showed by remarkable, punctual, clear staining in the cytoplasm of the mesothelial cells on these days (Fig. 4C–E). Sometimes the intense green signal even suppressed the blue color of the DAPI-labeled cell nuclei (Fig. 4C–E). Numerous punctate-like Bcl-2 signals could be identified in the long cytoplasmic extensions of the control mesothelial cells (Fig. 4F). The expression of Bcl-2 was considerably increased on the 3rd day after Freund's adjuvant treatment, compared to the control (Fig. 4G,K). However, Bcl-2 expression slightly decreased on the 5th day (Fig. 4H,K), during the recovery, Bcl-2 expression was still observed. Moreover, Bcl-2 signal intensity was further strengthened at the 8th and 11th day in the mesenteric mesothelial cells (Fig. 4I–K). Comparing the expressions of JNK1-JNK2 and Bcl-2 by statistical analysis, no significant difference was found. However, group-by-group comparison of JNK1-JNK1 or Bcl-2 showed several significant differences. Our statistical analysis revealed that there were significant differences in the expression intensities of JNK1-JNK2 between the 3rd and 8th (**p = 0.00164), 3rd and 11th (***p = 0.00039), and 5th and 11th days (*p = 0.02247). Regarding Bcl-2 signal intensities, we found significant differences between the following groups: CTR-D8 (**p = 0.0018), CTR-D11 (***p = 0.00043), D5-D8 (**p = 0.0047), D5-D11(**p = 0.00113) (Fig. 4K).

### The effect of BafA1 on mesothelial cells

#### Light microscopical studies

**Figure 5:**
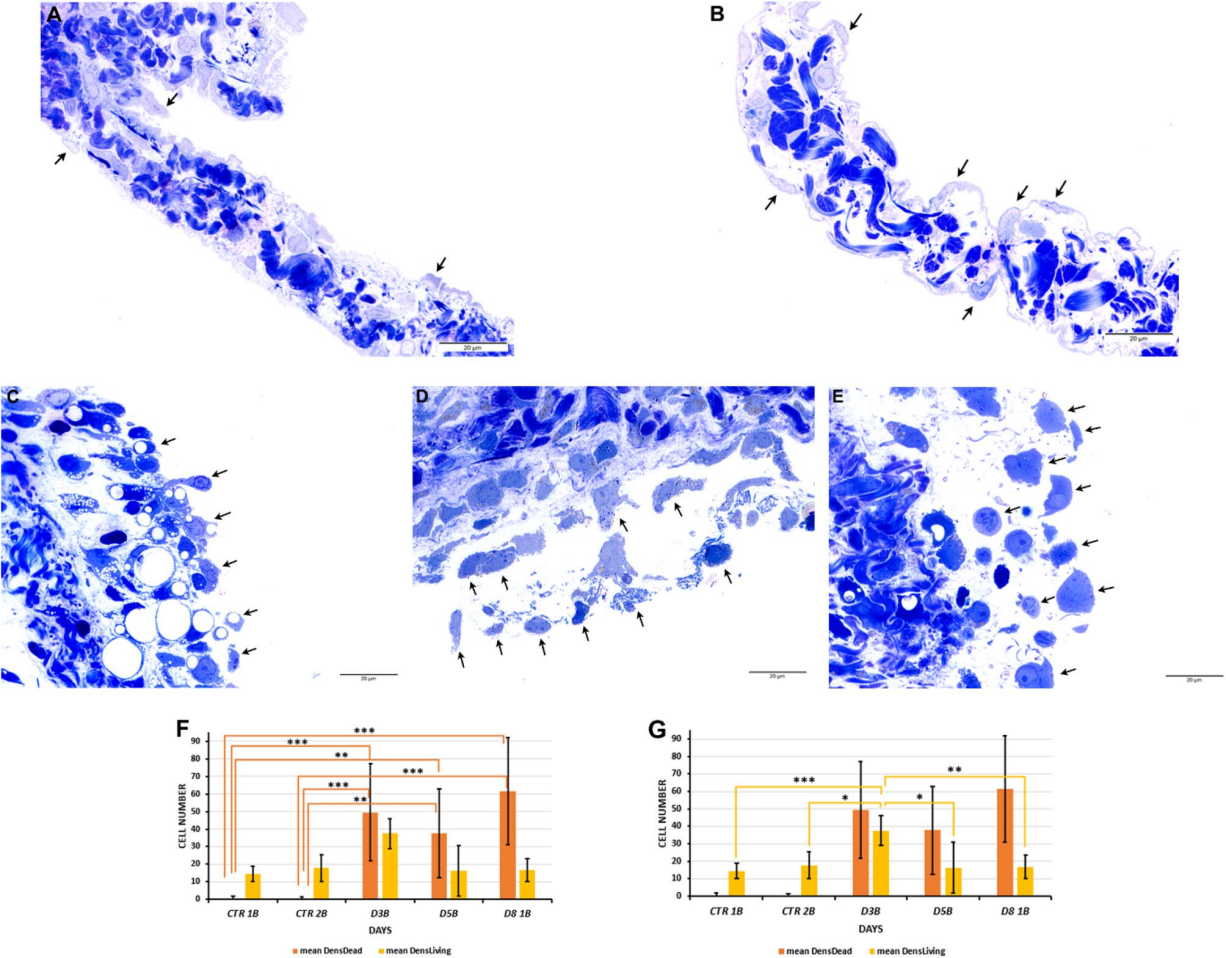
BafA1 treatment inhibited the regeneration and survival of mesothelial cells after Freund’s adjuvant injection (light microscopy). (**A**,**B**) Control mesothelial cells treated with BafA1 for 2 and 4 h did not show morphological alterations (CTR_1B_, CTR_1B_). (**C**) On day 3 of inflammation following Freund’s adjuvant injection, mesothelial cells from animals treated with BafA1 (4 h) became vacuolated and showed initial signs of cell death (D3_B_). (**D**) After 24 h of BafA1 treatment, on the 5th day of inflammation, mesothelial cells disintegrated, fragmented cytoplasmic residues could be seen in their environment (D5_B_). (**E**) After a single BafA1 treatment at day 4, on day 8 of inflammation, mesothelial cells were arranged in several rows on the surface of the connective tissue and their cell structure was barely recognizable (D8_1B_). (**F**,**G**) Statistical analyses about the number of dead versus living cells after BafA1 treatments in the mesentery (***p < 0.001; **p < 0.01; *p < 0.05) (arrows: mesothelial cells; data of the statistical analyses and cell numbers of the groups are presented in Supplementary Table S3). Bar = 20 μm.

BafA1 treatment alone did not alter the morphology of the mesentery even after 2 and 4 h (CTR_1B_, CTR_2B_). In these samples, mesothelial cells formed a unicellular, continuous layer on both surfaces of the mesentery. Their cytoplasm was thin, the cells were elongated, and they could be easily identified by their centrally located nuclei. Collagen fibers and various connective tissue cells were present as usual in the connective tissue of the mesentery (Fig. 5A,B).

On day 3 after Freund’s adjuvant injection, the inflamed mesothelial cells are rounded or cuboidal in shape. Although the cell junctions are disintegrated, they are still arranged in one row on each side of the connective tissue^6^. However, upon treatment with BafA1 for 4 h on day 3, the structure of inflamed mesentery was practically disintegrated, mesothelial cells became vacuolized, the chromatin was condensed in their nuclei, and the cells started to be disorganized. Connective tissue cells showed similar morphological changes (D3_B_) (Fig. 5C).

On day 5, mesothelial cells treated with Freund's adjuvant alone completely lose their squamous morphology and polarity, but their cytoplasm is intact and healthy, showing the typical morphological alterations caused by the inflammation^6^. When the animals were injected with BafA1 on day 4 of inflammation and after 24 h the mesentery was studied, the structure of the inflamed mesothelial cells changed dramatically (D5_B_). The BafA1 treated inflamed mesothelial cells were arranged in several rows, their cytoplasm was filled with smaller and larger vacuoles, cytoplasmic fragments and remnants of cells could be seen close to the surface of mesentery. Connective tissue cells showed similar morphological alterations, collagen fibers were arranged in dense, compact bundles (D5_B_) (Fig. 5D).

On day 8, mesenteric mesothelial cells not treated with BafA1 start to regenerate. Although most of the mesothelial cells are still rounded, they begin to reorganize into a single cell layer, and cell junctions begin to form between their long cytoplasmic extensions^6^. When BafA1 was injected to the abdominal cavity of the animals on day 4 of inflammation and the mesenteries was observed 4 days later (D8_1B_), mesothelial cells’ cytoplasm was fragmented, the cell organelles were practically unrecognizable, and only the rest of their disintegrating nuclei could be identified. No sign of autophagy and regeneration could be observed in the samples (D8_1B_) (Fig. 5E). The number of living and dead mesothelial cells was statistically analyzed in the control and inflamed mesentery after different BafA1 treatments. We found several significant differences between the examined groups (Fig. 5F,G). Regarding the number of dead cells, the non-inflamed groups treated only with BafA1 showed significant differences compared to the inflamed groups; their number increased significantly after the 3rd day of inflammation: CTR_1B_-D3_B_ ***p = 0.00072; CTR_1B_-D5_B_ **p = 0.00838; CTR_1B_-D8_1B_ ***p = 3.64 * 10^–5^; CTR_2B_-D3_B_ ***p = 0.00022; CTR_2B_-D5_B_ **p = 0.00318; CTR_2B_-D8_1B_ ***p = 9.16 * 10^–6^ (Fig. 5F). Regarding the number of living cells, we found significant differences between the following groups: CTR_1B_-D3_B_, ***p = 0.00031; CTR_2B_-D3_B_, *p = 0.01406; D3_B_-D5_B_, *p = 0.00131; D3_B_-D8_1B_, **p = 0.00556 (Fig. 5G).

#### Electron microscopical studies

**Figure 6:**
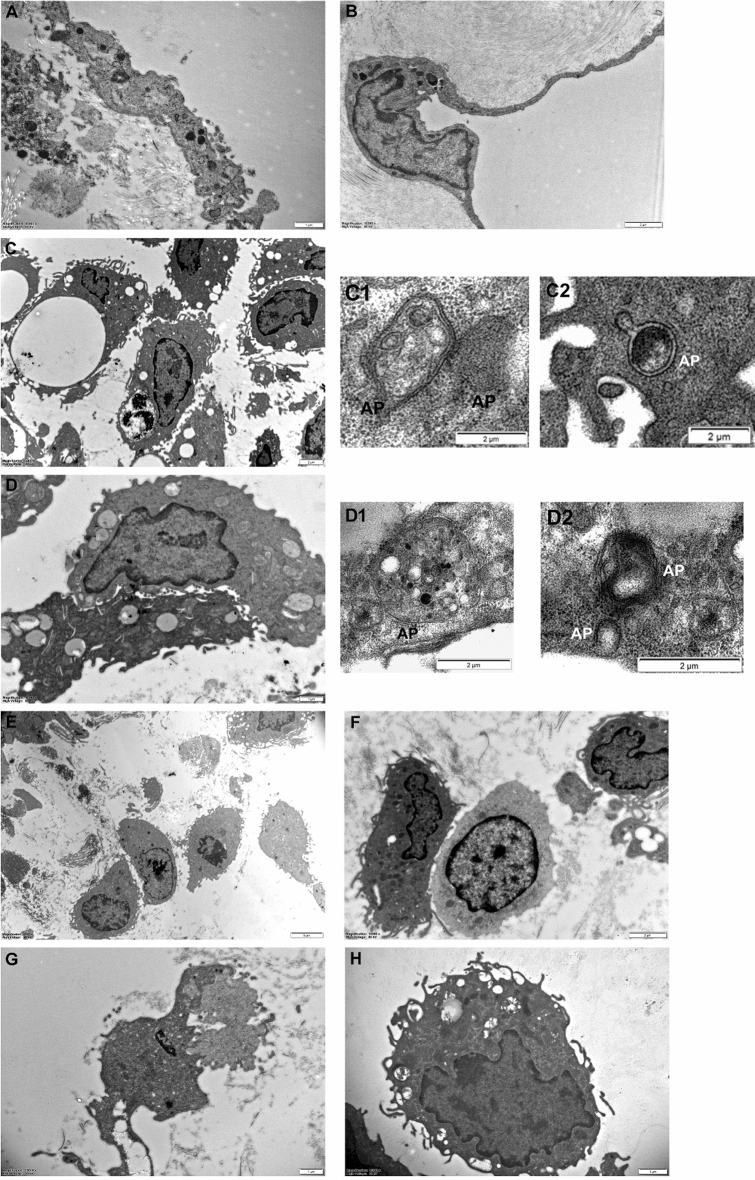
Ultrastructural signs of cell death in inflamed mesothelial cells after BafA1 treatments (electron microscopy). (**A**,**B**) Morphology of non-inflamed mesothelial cells treated with BafA1 (for 2 and 4 h, CRT_1B_, CTR_2B_) was like that of untreated mesothelial cells. (**C**) On day 3 of inflammation, mesothelial cells from animals treated with BafA1 (for 4 h, D3_B_) were highly vacuolated, and cytoplasmic fragments could be observed in their environment. (**C1**,**C2**) Only double-membrane-bounded autophagic vacuoles, immature autolysosomes could be seen in the mesothelial cells (D3_B_). (**D**) The mesothelial cells of animals treated previously with BafA1 for 24 h, showed detached bleb-like structures, and cell organelles with drastic morphological changes on the 5th day of inflammation (D5_B_). (**D1**,**D2**) Only young autophagosomes could be observed (D5_B_). (**E**–**H**) On day 8, no significant morphological differences could be detected between the groups in the series of single and combined treatments of BafA1 (E: D8_1B_, F: D8_2B_, G: D8_3B_, H: D8_4B_). (AP: autophagosome). Bar = 1, 2 μm.

No ultrastructural changes could be seen electron microscopically on mesenteric mesothelial cells treated with BafA1 for 2 and 4 h (CTR_1A_, CTR_1B_) comparing them with the control, non-treated and non-inflamed mesothelial cells. These findings indicate that BafA1 had no effect on the ultrastructure of mesothelial cells. They formed a continuous, thin, single cell epithelial layer on each side of the connective tissue and the cell junctions were intact. In their thin cytoplasm, the nuclei were dominant, and only a few cell organelles (endosomes, lysosomes, mitochondria, etc.) could be found. Furthermore, only a few young phagophores and non-acidifying autophagosomes, autolysosomes could be identified in the cytoplasm. Large number of caveolae were present on both the apical and basal plasma membranes; clathrin-coated vesicles were rarely seen. The structure and the organization of the connective tissue was like that of the healthy, non-treated mesentery (Fig. 6A,B).

In the mesenteric mesothelial cells of animals treated with BafA1 for 4 h on the 3rd day of inflammation (D3_B_), numerous vacuoles of various sizes appeared in the cells, and heterochromatin areas in their nuclei seemed to be more condensed and enlarged. The cell surface showed many short outfoldings, and detached cytoplasmic fragments could often be observed around the cells. Only early, double-membrane-bordered autophagosomes and immature autolysosomes could be identified in their cytoplasm (Fig. 6C1,C2). The connective tissue cells showed similar morphology (Fig. 6C).

The cytoplasm of the mesothelial cells isolated from animals treated with BafA1 for 24 h on day 5 of inflammation (D5_B_) were more vacuolated, the cell organelles were unrecognizable. Mitochondrial cristae were disintegrated, and the rough endoplasmic reticulum was considerably expanded. Cytoplasmic remnants detaching from the disintegrated cells could be detected. We found that apoptotic-like bleb formation was more characteristic. Only early autophagosomes could be identified in the cytoplasm (Fig. 6D1,D2). In contrast to the BafA1 treated cells, in the regenerating mesothelial cells that were not treated with BafA1, the number of autophagic vacuoles and mature autolysosomes are significantly increasing^6^. The chromatin was clamped to the inner surface of the nuclear membrane, which indicated a relatively high condensation and amorphous morphology (Fig. 6D).

Using light microscopy, no morphological differences could be found between single and multiple BafA1 treated animal groups on day 8 of inflammation. Only a few minor changes were detected between these groups with electron microscope. In summary, no sign of regeneration could be detected in mesothelial cells on day 8 of inflammation, when the animals were treated previously with BafA1. Instead, the cells showed specific morphological features of cell death. The cells shrank, the degree of vacuolation was increased; and large membrane-bordered fragments were detached from the cells. Their cytoplasm was shattered, and the bleb formation was prominent. Remnants of cell organelles and cytoplasmic debris often had a dense, homogeneous appearance. Cisterns of rough endoplasmic reticulum were swollen and dilated. Cell’ nuclei were disintegrated, and a dense marginal condensation of chromatin was usually significant (Fig. 6E–H).

### Detection of autophagic and apoptotic markers in BafA1 treated inflamed mesothelial cells

#### Detection of LC3B expression in BafA1 treated mesothelial cells

It is well-known that autophagy controls and assists in the degradation of unnecessary cell organelles and components^41,42^, thus it plays a crucial role in tissue reconstruction, remodeling, maintaining the cellular homeostasis^43^, inflammatory processes, immunity, and cellular stress responses^41,44,45^. Our previous morphological and morphometric results showed that during post-inflammatory regeneration the progressive autophagy is significant to restore the original squamous phenotype (MET)^6^.

To examine the effect of BafA1 on the autophagic flux, LC3B immunostainings and their statistical analysis were used. LC3B is a well-known and frequently used marker to illustrate the dynamics of autophagic activity. When autophagy is activated the cytosolic LC3B-I recruits to the APs’ membranes. As the APs fuse with lysosomes, the membrane-bound LC3B-II on the inner membranes is degraded while those presented on the external surface of the membranes become cytosolic again^46,47^.

**Figure 7:**
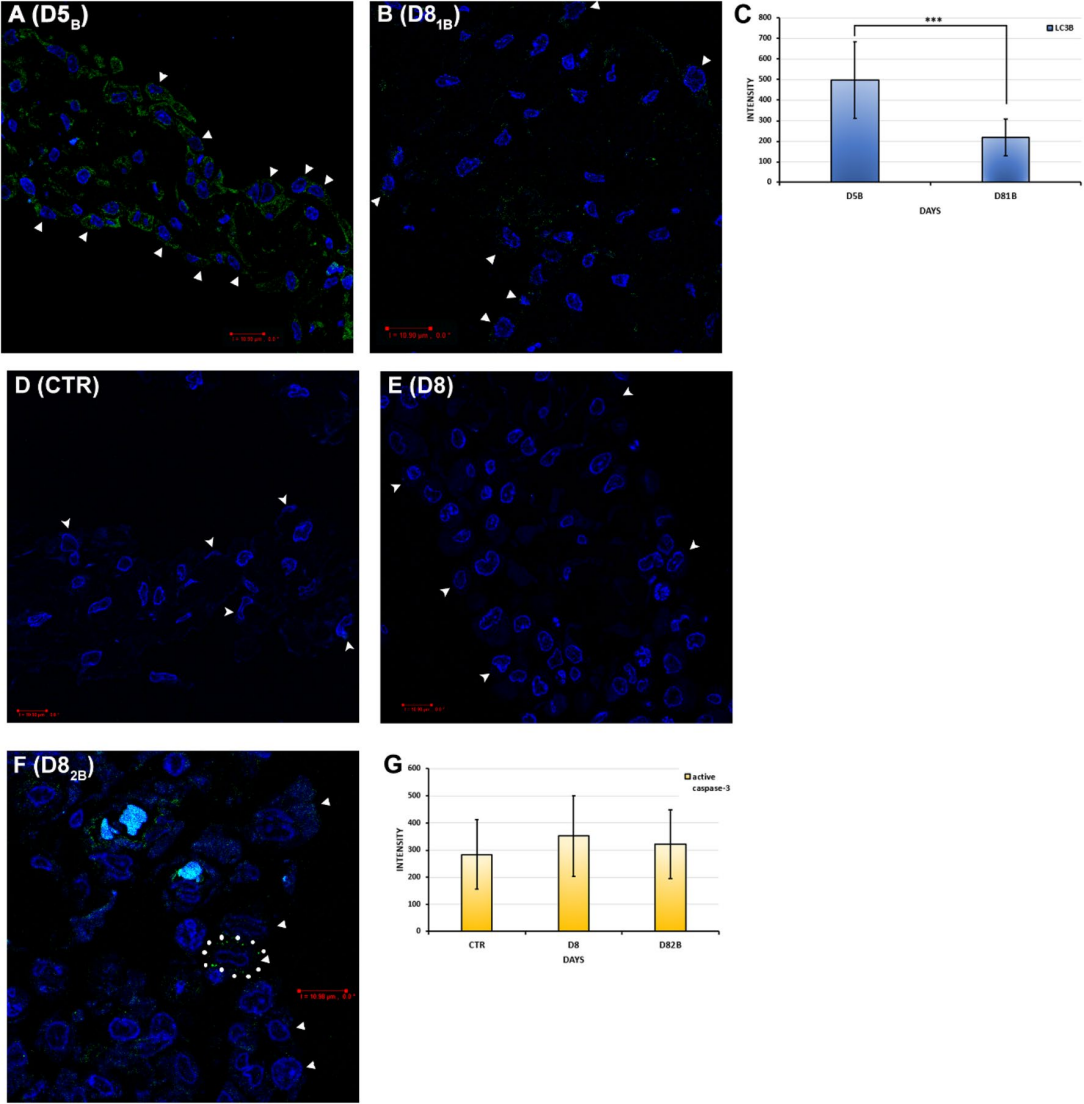
Effects of BafA1 treatment in healthy and inflamed mesothelial cells (IC on LC3B and active caspase-3 expressions). (**A**) Mesothelial cells of animals treated previously with BafA1 for 24 h showed weak and diffuse LC3B expression on day 5 of inflammation (D5_B_). (**B**) After BafA1 treatment at day 4, a barely detectable LC3B immunopositivity could be seen in the inflamed mesothelial cells on day 8 (D8_1B_). (**C**) Statistical analysis of LC3B signal intensities in mesothelial cells (***p < 0.001). (**D**,**E**) Caspase-3 activity could not be detected either in control cells or in inflamed cells without BafA1 treatment (D8). (**F**) In the inflamed mesothelial cells, treated four times with BafA1 (D8_2B_), only a few mesothelial cells showed active caspase-3 positivity (see the cell circled with dots). (**G**) Statistical analysis of active caspase-3 signal intensities in mesothelial cells. (There is no significant difference between the groups). (Arrowheads: mesothelial cells; blue: DAPI; green: (**A**,**B**) LC3B, (**D–F**) active caspase-3; data of the statistical analyses and cell numbers of the groups are presented in Supplementary Table S4). Bar ~ 10.80 µm.

Although mesenteries isolated from animals treated with BafA1 for 24 h on day 5 of inflammation (D5_B_) showed a weak LC3B immunopositivity, in the inflamed mesothelial cells treated once with BafA1 (D8_1B_), we could barely detect LC3B expression (Fig. 7A,B). Our statistical analysis revealed that there was a significant difference in the expression intensities of LC3B between the two treated groups (D5_B_-D8_1B_, ***p = 6.75*10^–7^). Furthermore, it showed that the LC3B expression was much lower than that of the previous proteins (Fig. 7C).

#### Detection of active caspase-3 expression in mesothelial cells

To reveal whether caspase-dependent or independent cell death processes take place in inflamed mesothelial cells after BafA1 treatment, immunostainings and statistical analysis were applied. Activation of caspases usually requires proteolytic cleavage next to an aspartate residue in their substrates^48^. Caspase-3 was selected as an effector caspase and a crucial executor caspase, that is either partially or totally responsible for the proteolytic cleavage of many intracellular proteins during apoptosis^49^.

Using antibody against active caspase-3 (Asp175), we could not detect caspase-3 activity in either the control, healthy mesothelial cells (CTR), or at the 8th day of inflammation without BafA1 treatment (D8) (Fig. 7D,E). In the inflamed mesentery, treated four times with BafA1 (D8_2B_), just a few mesothelial cells showed active caspase-3 immunopositivity, but most of the cells were negative (Fig. 7F). Comparing the expressions of active caspase-3, no significant difference was found between the three investigated groups (Fig. 7G).

## Discussion

In our previous studies, we confirmed that during the Freund’s adjuvant induced inflammation, rat’s mesenteric mesothelial cells losing their characteristic epithelial feature differentiate into mesenchymal, macrophage-like cells (EMT II)^1–6^. If there is no further Freund’s adjuvant injection, after the peak time of inflammation the regeneration (MET) starts, and the inflamed mesothelial cells regain their original simple squamous phenotype by the 11th day^6^.

EMT and MET can be controlled by several signaling molecules. The question arises as to which factors and signaling cascades are activated during the autophagy-mediated regeneration (MET) in our model system. TGF-β is known to induce EMT, however BMP proteins are likely to induce MET^15,26–28^, and several studies have shown, that BMP signals and their signaling effects are important in repairing and regenerating injured tissues^50–52^. Therefore, we were interested in the expression of BMP proteins after Freund’s adjuvant induced inflammation. Numerous experiments and literature data support that BMP7 is one of the most potent inducer of MET and an inhibitor of EMT^27,28,53^. Less is known whether BMP4 is also able to induce MET^54^. In fact, heterodimers of these two factors (BMP4/7) are known to be much more effective than their homodimers^55^. Considering this, we studied the time dependent alterations of BMP4/BMP7 production and in correlation with these the BMPR1A and 2 expressions of mesothelial cells during inflammation and regeneration.

Our results showed that the intracellular expression of BMP4 could already be detected in control mesenteric mesothelial cells, by the time of inflammation (D3, D5), its expression intensity was increased, and still high as the regeneration started. It is important to highlight that, in addition to mesothelial cells, connective tissue cells also expressed a considerable amount of BMP4. Comparing the expression of BMP4 with BMP7 we found that BMP7 was also expressed by mesothelial cells. However, the intracellular amount of BMP7 was lower in the mesothelial cells, its expression was gradually increased in the peritoneal cavity during Freund's adjuvant induced inflammation and reached the maximum on day 8, when the recovery started. Additionally, the expression differences of BMP4 and BMP7 during recovery from Freund’s adjuvant caused inflammation could endorse our previous hypothesis that the mesothelial synthesis of BMP4 was upregulated in the regeneration of mesothelial cells (MET) besides the already described effect of BMP7.

Active BMP ligands induce signaling pathways through serine/threonine kinase receptors (BMPR-I and BMPR-II)^12^. There are several subtypes of these receptors. In our system we focused on BMPR1A and BMPR2. It is known that BMP6 and BMP7 interact with type II and recruit type I receptors, whereas BMP2 and BMP4 preferentially bind to type I and recruit type II receptors^16,17^. Our results proved that BMPR1A was expressed even in control mesothelial cells and on different days of inflammation. Regarding BMPR2, we found that it was also expressed in the control cells, and its expression was significantly increased after the Freund’s adjuvant injection. Binding to preformed type I and type II serine/threonine kinase receptor complexes versus BMP-induced receptor recruitment can stimulate different pathways^17^. Based on this, we concluded that the production of BMP4 and BMP7 by mesothelial cells and the significant expression of BMPR1A and BMPR2 induced special signaling mechanism during the recovery of the mesothelial cells simultaneously with the intense autophagy.

BMPs can act via both canonical and non-canonical pathways leading to a specific cellular output^16,17^. Nowadays it is generally accepted that, besides of the several roles of BMPs in various cell mechanisms, they can also take part in promoting autophagy through SMAD-independent pathway^56^. For example, the BMPR1A-TAK1-p38MAPK signaling pathway is a known non-canonical transduction pathway of BMPs, which plays a critical role in club cell regeneration^57^. Therefore, we were curious about which BMP-induced signaling pathway dominates in our model system.

In the canonical signaling pathway, BMPs induce signal transduction cascade through the SMAD1/5/8-SMAD4 pathway^17,18^. In addition to SMADs, several non-canonical pathways have been identified for BMP factors^17^. The best-established route that BMPs regulate is the TAK1-p38 pathway through recruitment and ubiquitylation of TNF receptor-associated factor-6 (Traf6) by activated receptor complexes^16,18^. It is well-known, that TAK1 is a central modulator of cell death as well as survival. The loss of TAK1 activity results in apoptosis in numerous tissue types^19^. There are also data, that TAK1 can activate AMPK-dependent cytoprotective autophagy^58^. Activated TAK1 phosphorylates and activates MAPKKs leading to stimulation of MAPKs such as ERK, p38 and JNK^19^. Consequently, the downstream targets of JNKs control expression of pro-apoptotic or anti-apoptotic genes *Bax* (Bcl2-associated X protein) and *Bcl-2*^21^. Additionally, JNK dependent phosphorylation of c-Jun/c-Fos enhances transcriptional activity of Beclin1. On the other hand, constitutively activated JNK1 phosphorylates Bcl-2 at Thr^69^, Ser^70^ and Ser^87^ amino acid residues, which dissociates Bcl-2 from Beclin1 and constitutes for the assembly of the Beclin1-related PI3K III complexes inducing autophagy^21^.

Regarding EMT and MET, we examined the expression of p-SMAD1/5 representing the canonical and the TAK1-JNK-Bcl-2 signaling axis representing the non-canonical pathway in mesothelial cells. The expression of p-SMAD1/5 was low and did not increase dramatically in the days of inflammation and regeneration. On the other hand, the expression of TAK1 clearly increased during the days of inflammation and the JNK1-JNK2 kinases were also intensely expressed with the beginning of the regeneration. Studying the Bcl-2 expression the results showed that its expression was considerably increased on the 3rd day of Freund's adjuvant induced inflammation and further strengthened in the days of the recovery. Bcl-2 intensity could be detected with definitive, dot-like signals in the cytoplasm of the mesenteric mesothelial cells. The prominent expression of JNK kinases and Bcl-2 may contribute to the activation of Beclin-1 which aids the induction of autophagic processes. Our results verify that the BMPs-induced SMAD-independent signaling pathway was dominant over the canonical route in our model system. Activation of the TAK1-JNK-Bcl-2 signaling axis coincided in time with the initiation of protective autophagy and regeneration of mesothelial cells.

Our previous morphological and morphometric results show that during post-inflammatory regeneration the progressive autophagy is significant to restore the original squamous phenotype (MET)^6^. To prove the importance of autophagy in the recovery of inflamed mesothelial cells, we studied the regeneration in the presence of BafA1 that is one of the most commonly used and well-proven inhibitor of autophagy. It prevents fusion between autophagosomes and lysosomes, and inhibits the acidification of endosomes and lysosomes, disrupting the dynamics of the autophagic flux^59–61^.

In the present study we have shown with light and electron microscopic observations that BafA1 treatments for 2 and 4 h alone had no effect on mesothelial cells’ morphology. When inflamed mesothelial cells were treated with BafA1 on day 3 or 4 after Freund’s adjuvant injection, prominent alterations could be detected in their morphology. Numerous vacuoles of varying sizes, detached cytoplasmic fragments, disintegrated, and expanded cellular organelles could be observed in the cytoplasm of the mesothelial cells in both groups. These results show that by the time, the BafA1 treated, inflamed mesothelial cells undergo more and more characteristic morphological changes, apoptotic alterations as well as signs of other types of cell death could be detected inside of their cytoplasm^62–65^. On day 8, it was clear that when the autophagy was inhibited by different BafA1 treatments, mesothelial cells have died. No signs of regeneration could be observed. In the dying cells’ nuclei and nucleoli disintegrated, and marginally condensed chromatin stock was spectacular and conspicuous. The most prominent ultrastructural alterations were found in D8_2B_ and D8_3B_ groups, at that time when in cells not treated with BafA1, an intense autophagy is already initiated^6^. From these data, we can conclude that autophagy plays an essential role in the post-inflammatory recovery of mesothelial cells (MET), and consequently, by inhibiting this process, inflamed mesothelial cells die. To support our morphological observations, we performed a statistical analysis to count the living versus dead cells after BafA1 treatments in the mesentery. We found several significant differences between the examined groups. Overall, the number of dead cells increased over time compared to the number of living cells.

Examining the effect of BafA1 on the autophagic flux in the mesenteries isolated from animals treated with BafA1, although a weak LC3B immunopositivity was detected on day 5, but after more days, LC3B expression was barely seen. These results proved that LC3B expression could only be observed when there were some young, double-membrane-bordered APs in the mesothelial cells on the 5th day of inflammation with a small amount of LC3B-I in the cytoplasm and LC3B-II on their surfaces. Furthermore, on day 8 after BafA1 treatment, the cytosolic LC3B-I was no longer expressed, and there were no autophagic organelles with membrane-bound LC3B-II.

To answer the question whether caspase-dependent or independent cell death processes take place in inflamed mesothelial cells when the autophagy is inhibited, immunostainings were applied in healthy, Freund’s adjuvant and combined, Freund’s adjuvant and BafA1 treated mesenteries. Caspases are cysteine proteases, whose role is to execute programmed cell death, like apoptosis. Caspases exist as inactive zymogens in cells and undergo a regulated cascade of catalytic activation^66^. Caspase-3 is an effector caspase, which is responsible for the proteolytic cleavage of many key proteins such as protein kinase C (PKCδ) or poly(ADP-ribose) polymerase (PARP)^49,67^. It is known that a specific initiator caspase can mediate the intrachain cleavage of an effector caspase resulted in its activation^48^. This proteolytic cleavage generally occurs next to an aspartate residue^66^. As the immunocytochemical results showed, no caspase-3 activity was detected either in control or only Freund’s adjuvant treated, inflamed mesothelial cells. In the BafA1 treated, inflamed mesentery, only a few mesothelial cells showed active caspase-3 immunopositivity, but most of them were negative. These data strongly support Yan et all’s results on hepatocellular carcinomas, in which they conclude that BafA1 promotes caspase-independent cell death^68^. With these data, we could demonstrate the essential role of autophagy in the regeneration of mesothelial cells.

**Figure 8:**
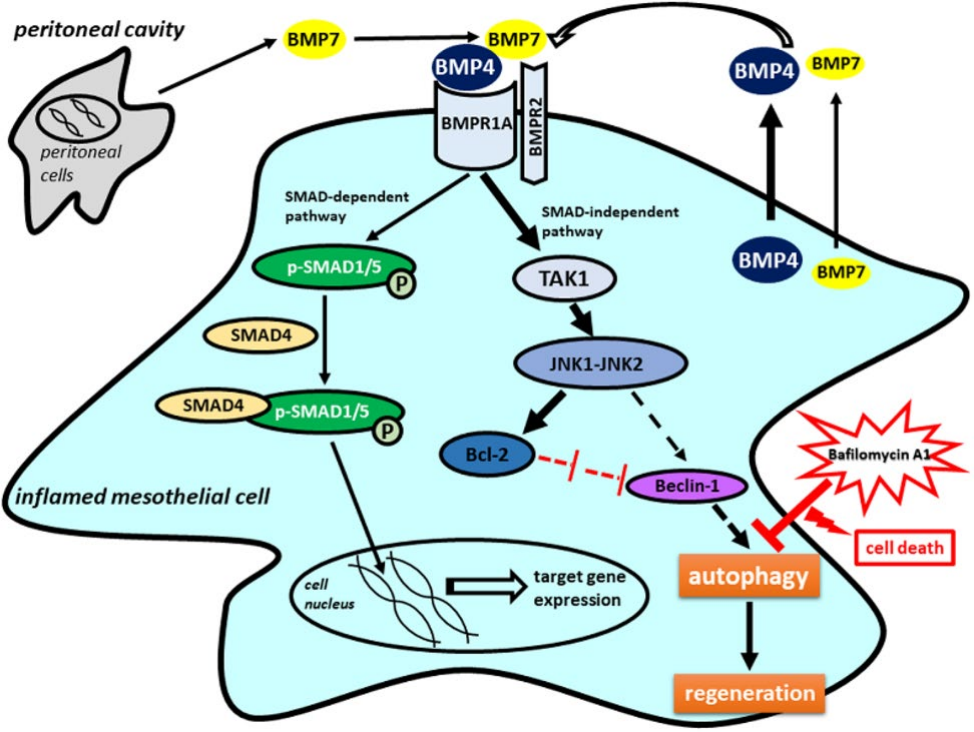
Expression of BMPs and their signaling pathways in the post-inflammatory recovery of mesothelial cells accompanied with protective autophagy. BMP4 and BMP7 were expressed by mesothelial cells. Other peritoneal cells also participated in BMP7 production. We presume that the more effective BMP4 and 7 heterodimers stimulated signaling through an auto/paracrine way in the inflamed mesothelial cells. The BMP specific BMPR1A and BMPR2 were also expressed in mesenteric mesothelial cells. BMPR1A induced SMAD-independent signal transduction through TAK1-JNK-Bcl-2 pathway, which was dominant over the canonical route represented by p-SMAD1/5 and was upregulated during the autophagy-mediated regeneration of inflamed mesothelial cells. Presumably, JNK signaling may indirectly (e.g., via Bcl-2) contribute to the activation of Beclin-1, which plays an important role in inducing autophagy. BafA1 treatment prevented the regeneration and induced caspase-3 independent cell death in inflamed mesothelial cells proving the regenerative role of autophagy after inflammation. (Bold arrows indicate the dominant SMAD-independent pathway and the significant expression of BMP4 versus BMP7 in mesothelial cells. The direct regulations are shown by continuous arrows, the indirect regulations are indicated by dashed arrows.).

Overall, we concluded that the regeneration of mesothelial cells (MET) was accompanied by the upregulated expression of BMP factors. Mesothelial cells expressed both BMP4 and BMP7 which in the presence of BMPR1A and BMPR2 induced signaling processes by an auto-paracrine regulation. Even though BMP7 was expressed in a smaller amount by the mesothelial cells than BMP4, we suppose that BMP7 was secreted into the peritoneal cavity by other types of cells as well, and certainly enhanced the effect of BMP4 in heterodimer form. We proved that the TAK1-JNK-Bcl-2 SMAD-independent signaling pathway was activated after Freund’s adjuvant induced inflammation and dominated during the days of regeneration, simultaneously with the initiation of the cytoprotective autophagy in mesothelial cells. To prove the importance of autophagy in the recovery process, we applied BafA1 treatment. Our results showed that inflamed mesothelial cells treated with BafA1 were not able to regenerate, and they died with caspase-3 independent cell death when autophagy was inhibited. These data strongly confirm that autophagy is indispensable in the post-inflammatory regeneration of mesothelial cells (MET) (Fig. 8).

## Supplementary Information

Supplementary Information.

## Data Availability

The datasets used and/or analyzed in this study are included in the manuscript and in the Supplementary file, further inquiries can be directed to Viktória Zsiros.
