# Supplementary material for: BMP-induced non-canonical signaling is upregulated during autophagy-mediated regeneration in inflamed mesothelial cells
Source: Sci Rep. 2023 Jun 27;13:10426. doi: 10.1038/s41598-023-37453-x (PMC10300029; doi:10.1038/s41598-023-37453-x)
Supplement: Supplementary file 1 — Supplementary Information. [file 41598_2023_37453_MOESM1_ESM.pdf]

“BMP-induced non-canonical signaling is upregulated during autophagy-mediated regeneration in inflamed mesothelial cells”

Viktória Zsiros[1]*, Nikolett Dóczi[1], Gábor Petővári[2], Alexandra Pop[1], Zsófia Erdei[1], Anna Sebestyén[2] and Anna L. Kiss [1].

- During WB analysis for detecting the immune responses we could not visualize the loading control (β-actin) together with the test protein (BMPR1A and BMPR2), but the same concentration and amount of samples were placed in the gels running in parallel for each electrophoresis.
- The peritoneal fluid type sample does not have an accepted loading control (in the case of BMP7).
- The WB images in our manuscript were only cropped from the original membranes and slightly lightened.

Supplementary Fig. S1

BMP7 expression in the peritoneal fluid.

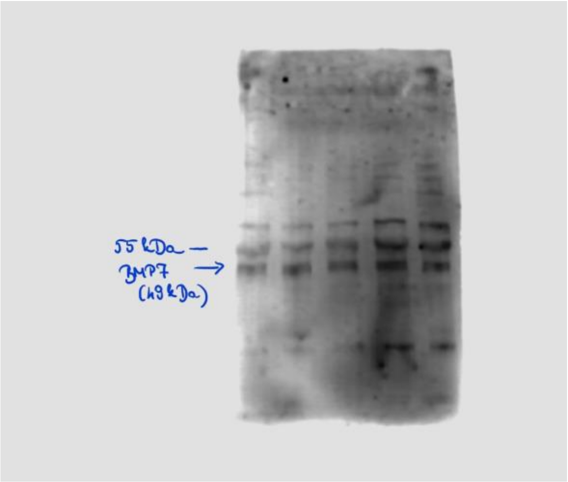

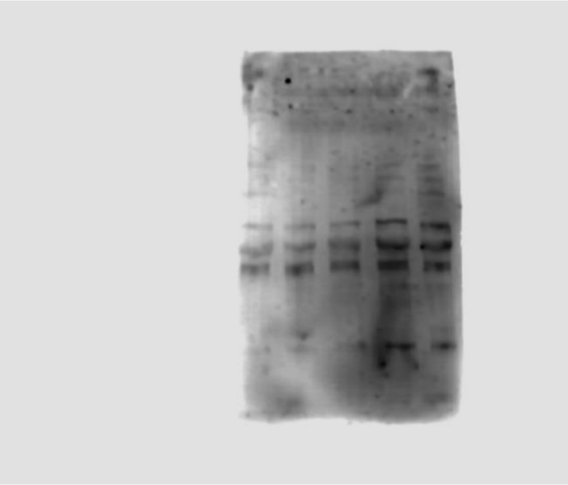

- original blot without labelings –

The edge of the membrane is well visible.

Supplementary Fig. S2A

BMPR1A expression in the mesothelial cell lysate.

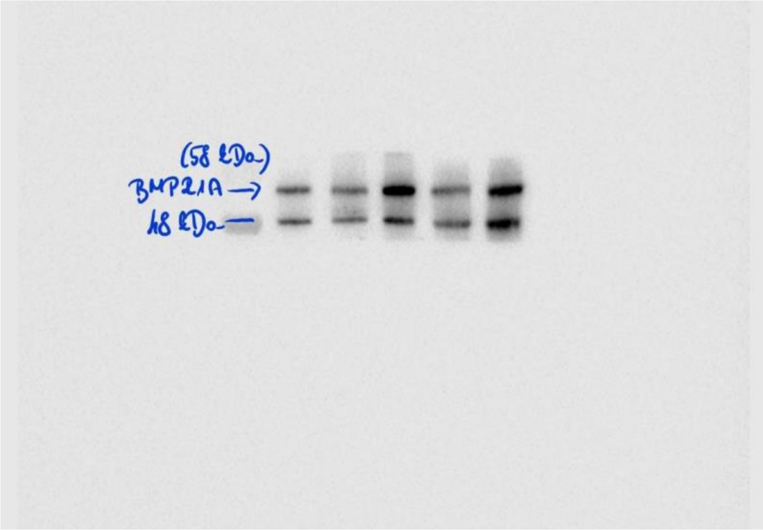

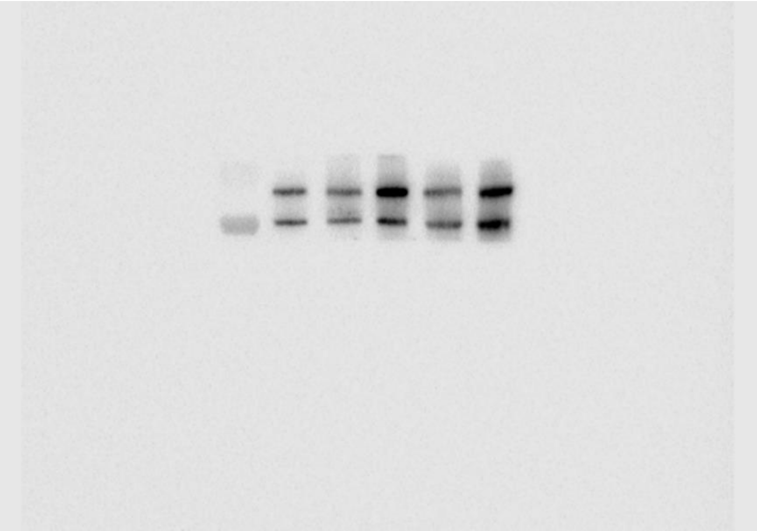

- original blot without labelings -

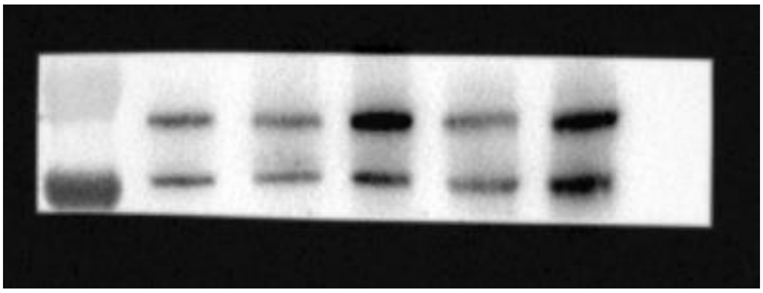

The edge of the membrane is unfortunately not visible on the first 2 images, but this is how it was developed. In this case, the detection was made with an exposure time of 14 seconds, so the edge of the membrane was not "burnt" into the image. Only the part of the membrane that fell into the measurement range was detected. For the avoidance of doubt, we have attached the image of the original blot merged with the protein ladder.

Supplementary Fig. S2B

β-actin expression in the mesothelial cell lysate.

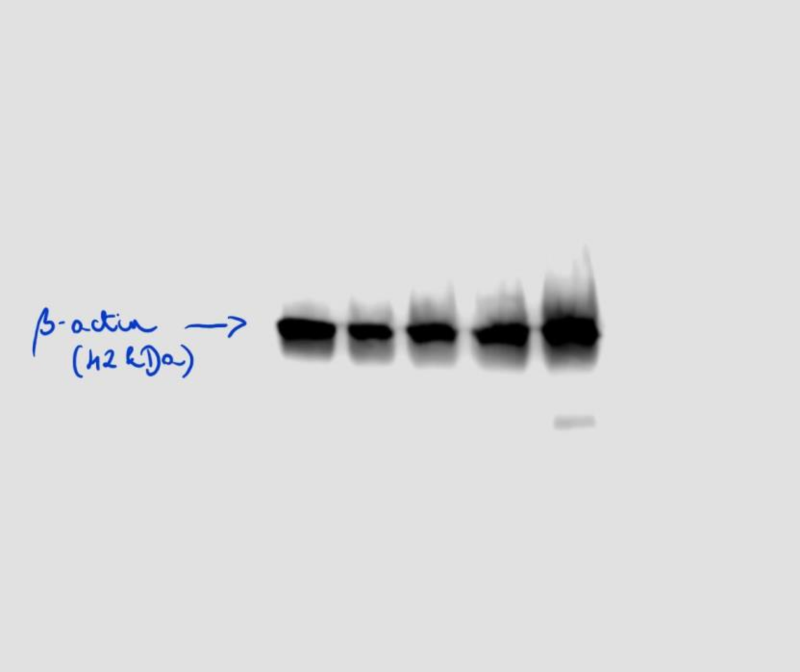

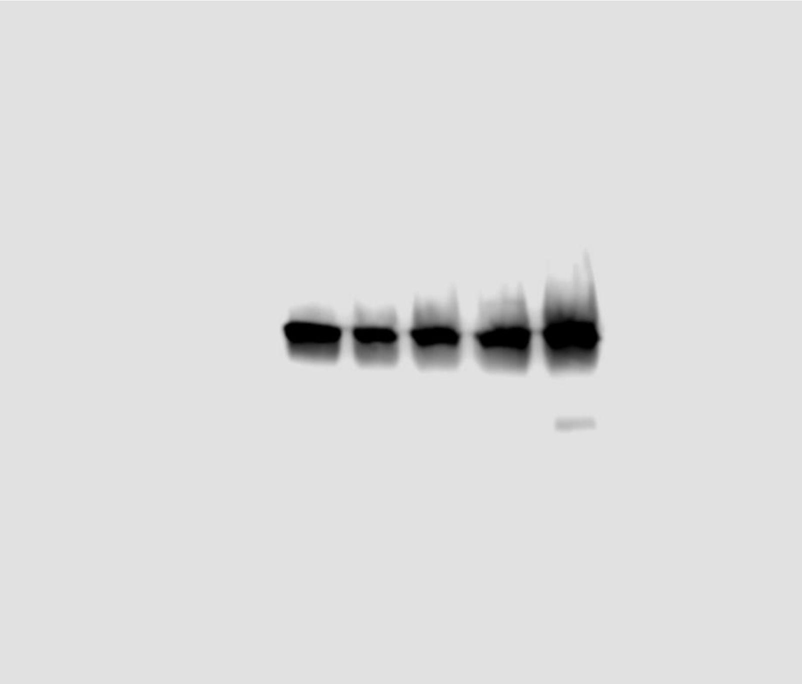

- original blot without labelings –

The edge of the membrane is unfortunately not visible, but this is how it was developed. In this case, the protein ladder was not visible, even though chemiluminescent HRP detection reagent was applied on the entire membrane.

Supplementary Fig. S2C

BMPR2 expression in the mesothelial cell lysate.

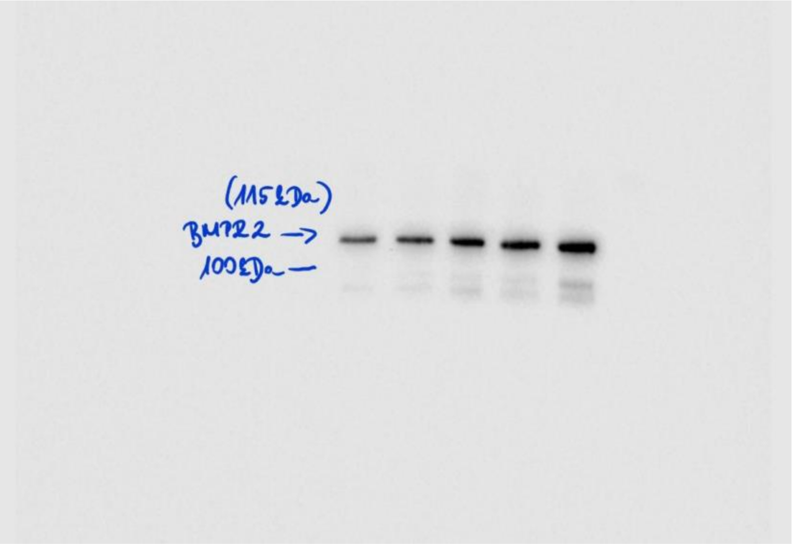

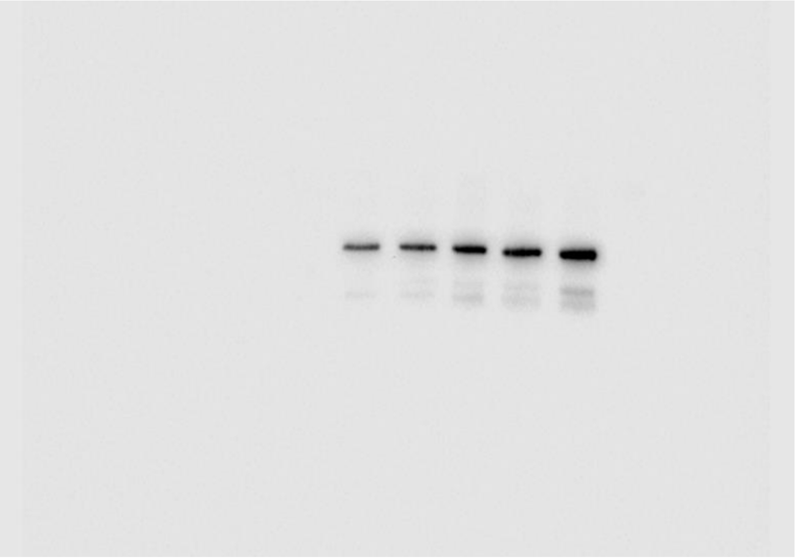

- original blots without labelings -

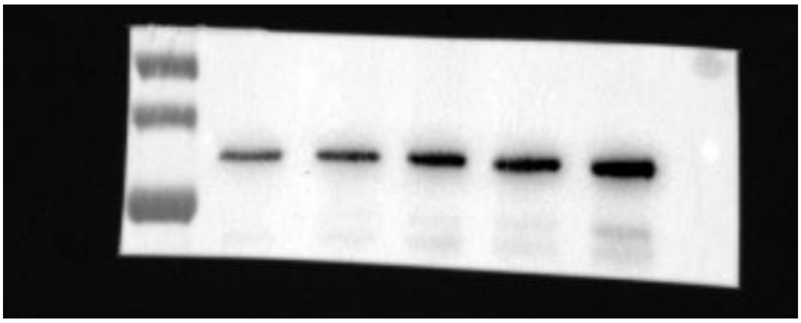

The edge of the membrane is unfortunately not visible on the first 2 images, but this is how it was developed. In this case, the detection was made with an exposure time of 14 seconds, so the edge of the membrane was not "burnt" into the image. Only the part of the membrane that fell into the measurement range was detected. For the avoidance of doubt, we have attached the image of the original blot merged with the protein ladder.

Supplementary Fig. S2D

β-actin expression in the mesothelial cell lysate.

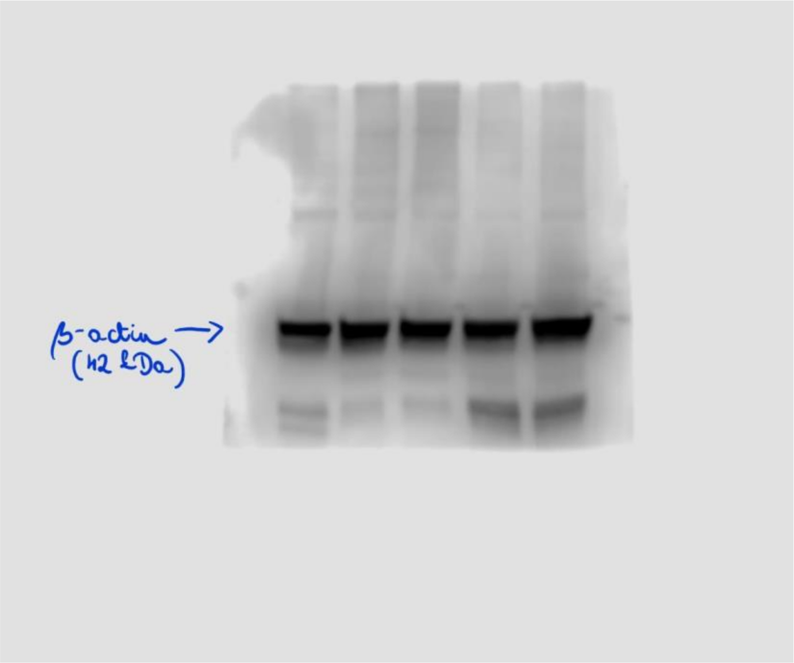

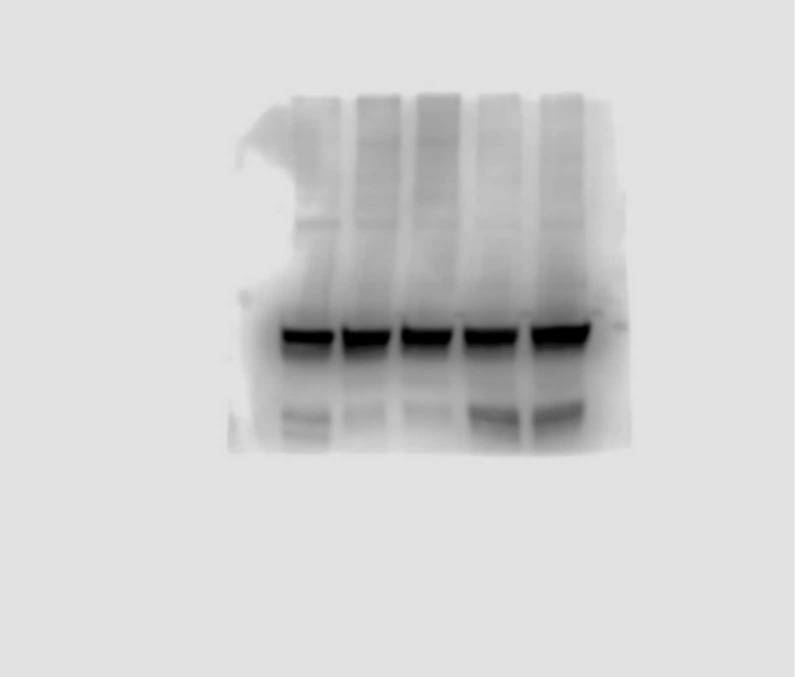

- original blot without labelings –

The edge of the membrane is well visible. In this case, the protein ladder was not visible, even though chemiluminescent HRP detection reagent was applied on the entire membrane.

Supplementary Table S1

Data for the statistical analyses about the signal intensity of BMP4, BMP7, BMPR1A, BMPR2, p-SMAD1/5 and TAK1.

Supplementary Table S1A: Means and standard deviations of BMP4 signal intensity.

Day	n	Mean	Std
CTR	44	2137.881	1157.266
D3	55	3689.148	1749.256
D5	36	3817.335	1590.639
D8	75	2256.665	1108.508
D11	44	1378.508	623.7746

Supplementary Table S1B: Means and standard deviations of BMP7 signal intensity.

Day	n	Mean	Std
CTR	42	347.2614	184.8314
D3	53	1465.268	729.3439
D5	38	1268.642	631.8876
D8	35	881.1411	472.808
D11	45	1139.953	733.9173

Supplementary Table S1C: Means and standard deviations of BMPR1A signal intensity.

Day	n	Mean	Std
CTR	23	1545.803	716.8414
D3	51	943.1228	661.2007
D5	66	1010.739	519.64
D8	29	1449.272	547.4728
D11	44	1038.361	426.2671

Supplementary Table S1D: Means and standard deviations of BMPR2 signal intensity.

Day	n	Mean	Std
CTR	31	833.8444	422.0972
D3	46	3965.856	1680.568
D5	39	6519.156	3447.223
D8	43	1993.855	1142.683
D11	46	2668.517	1232.526

Supplementary Table S1E: Means and standard deviations of p-SMAD1/5 signal intensity.

Day	n	Mean	Std
CTR	25	168.9739	87.74257
D3	41	232.5793	89.51092
D5	49	357.7025	153.0749
D8	31	167.4961	94.45943

D11	28	67.86782	50.71088

Supplementary Table S1F: Means and standard deviations of TAK1 signal intensity.

Day	n	Mean	Std
CTR	25	740.8482	343.451
D3	33	3626.746	1709.019
D5	45	1250.116	680.7791
D8	27	1531.861	526.917
D11	27	274.1033	161.6938

Supplementary Table S1G: Code numbers and their p-values in control (CTR) and inflamed groups (D3, D5, D8, D11).

**BMP4**	**1**
**BMPR1A**	**2**
**BMP7**	**3**
**BMPR2**	**4**
**p-Smad1/5**	**5**
**TAK1**	**6**

CTR:

**Pairwise comparison**		**D1**	**D2**	**low limit**	**difference**	**up limit**	**p-values**
		1	2	-25.7249	14.59783	54.92054	0.907408857
		1	3	69.94345	103.75	137.5566	8.02E-18
		1	4	17.47039	54.21774	90.9651	0.000376075
		1	5	93.0808	132.33	171.5792	7.56E-22
		1	6	19.9608	59.21	98.4592	0.000248088
		2	3	48.50122	89.15217	129.8031	5.92E-09
		2	4	-3.50763	39.61992	82.74746	0.092896031
		2	5	72.45395	117.7322	163.0104	1.39E-12
		2	6	-0.66605	44.61217	89.89039	0.056210361
		3	4	-86.6395	-49.5323	-12.425	0.00197669
		3	5	-11.0063	28.58	68.16634	0.309951364
		3	6	-84.1263	-44.54	-4.95366	0.016927437
		4	5	35.98669	78.11226	120.2378	1.88E-06
		4	6	-37.1333	4.992258	47.11783	0.999422485
		5	6	-117.445	-73.12	-28.7951	3.81E-05

D3:

**Pairwise comparison**	**D1**	**D2**	**low limit**	**difference**	**up limit**	**p-values**

		1	2	72.42581	117.1226	161.8195	8.76E-13
		1	3	40.12267	84.37976	128.6368	8.22E-07
		1	4	-55.5253	-9.58538	36.35459	9.91E-01
		1	5	132.2882	179.729	227.1699	0.00E+00
		1	6	-49.2222	1.406061	52.03435	1.00E+00
		2	3	-77.8436	-32.7429	12.35788	3.04E-01
		2	4	-173.461	-126.708	-79.9547	1.06E-13
		2	5	14.37753	62.60641	110.8353	2.96E-03
		2	6	-167.084	-115.717	-64.3492	1.93E-09
		3	4	-140.298	-93.9651	-47.6321	1.11E-07
		3	5	47.52767	95.34929	143.1709	1.97E-07
		3	6	-133.959	-82.9737	-31.9885	5.17E-05
		4	5	139.9312	189.3144	238.6976	0.00E+00
		4	6	-41.4613	10.99144	63.44414	9.91E-01
		5	6	-232.095	-178.323	-124.551	3.47E-21

D5:

Pairwise comparison		D1	D2	low limit	difference	up limit	p-values
		1	2	64.79986	111.4167	158.0335	1.29E-10
		1	3	39.73727	92.06579	144.3943	7.91E-06
		1	4	-69.9434	-17.9423	34.05881	9.23E-01
		1	5	140.7084	190.0969	239.4854	0.00E+00
		1	6	43.37376	93.68333	143.9929	1.66E-06
		2	3	-65.167	-19.3509	26.46527	8.35E-01
		2	4	-174.801	-129.359	-83.9171	3.51E-15
		2	5	36.25306	78.68027	121.1075	1.87E-06
		2	6	-61.2293	-17.7333	25.76262	8.55E-01
		3	4	-161.293	-110.008	-58.7235	1.42E-08
		3	5	49.39766	98.03115	146.6646	1.36E-07
		3	6	-47.9511	1.617544	51.18614	1.00E+00
		4	5	159.7582	208.0392	256.3203	0.00E+00
		4	6	62.4028	111.6256	160.8485	1.45E-09
		5	6	-142.868	-96.4136	-49.9594	4.89E-08

D8:

Pairwise comparison		D1	D2	low limit	difference	up limit	p-values
		1	2	0.186938	43.26943	86.35191	4.83E-02
		1	3	50.65507	90.98667	131.3183	1.82E-09
		1	4	-23.4583	14.22853	51.91538	8.91E-01
		1	5	105.0918	147.6533	190.2149	0.00E+00
		1	6	-7.76489	36.45333	80.67156	1.75E-01
		2	3	-1.75604	47.71724	97.19052	6.61E-02
		2	4	-76.3829	-29.0409	18.30112	5.00E-01

		2	5	53.0765	104.3839	155.6913	9.92E-08
		2	6	-59.5059	-6.81609	45.87375	9.99E-01
		3	4	-121.611	-76.7581	-31.905	1.59E-05
		3	5	7.64639	56.66667	105.6869	1.27E-02
		3	6	-104.999	-54.5333	-4.06794	2.53E-02
		4	5	86.55639	133.4248	180.2932	3.50E-15
		4	6	-26.1531	22.22481	70.60266	7.80E-01
		5	6	-163.465	-111.2	-58.9353	1.95E-08

D11:

Pairwise comparison		D1	D2	low limit	difference	up limit	p-values
		1	2	-16.4464	24.68182	65.81001	5.25E-01
		1	3	-17.2905	23.60859	64.50765	5.69E-01
		1	4	-92.0967	-51.418	-10.7393	4.27E-03
		1	5	81.50137	128.1364	174.7714	4.26E-14
		1	6	52.33767	99.49747	146.6573	2.68E-08
		2	3	-41.9723	-1.07323	39.82584	1.00E+00
		2	4	-116.778	-76.0998	-35.4211	1.45E-06
		2	5	56.81955	103.4545	150.0895	3.70E-09
		2	6	27.65586	74.81566	121.9755	9.00E-05
		3	4	-115.474	-75.0266	-34.5796	1.86E-06
		3	5	58.09473	104.5278	150.9608	1.99E-09
		3	6	28.92878	75.88889	122.849	6.04E-05
		4	5	133.3153	179.5543	225.7934	0.00E+00
		4	6	104.1472	150.9155	197.6838	6.72E-20
		5	6	-80.671	-28.6389	23.39322	6.19E-01

Supplementary Table S2

Statistical analysis of WB densitometry data on BMP7, BMPR1A and BMPR2 expressions.

Supplementary Table S2A: Code numbers for each group.

**CTR**	**1**
**D3**	**2**
**D5**	**3**
**D8**	**4**
**D11**	**5**

Supplementary Table S2B: Means, standard deviations and p-values of BMP7 expression in the peritoneal fluid.

BMP 7 (PF)	**CTR**	**D3**	**D5**	**D8**	**D11**
**average**	1	1.438310178	1.5886145	1.708874743	1.111685969

**standard deviation**	0	0.134976936	0.302992087	0.222208569	0.252264099

**Pairwise comparison**		**D1**	**D2**	**low limit**	**difference**	**up limit**	**p-values**
		1	2	-1.00585	-0.43831	0.12923	0.156574
		1	3	-1.15615	-0.58861	-0.02107	0.041357
		1	4	-1.27641	-0.70887	-0.14133	0.014116
		1	5	-0.67923	-0.11169	0.455854	0.963212
		2	3	-0.71784	-0.1503	0.417236	0.900965
		2	4	-0.8381	-0.27056	0.296975	0.546007
		2	5	-0.24092	0.326624	0.894164	0.378694
		3	4	-0.6878	-0.12026	0.44728	0.952479
		3	5	-0.09061	0.476929	1.044468	0.112129
		4	5	0.029649	0.597189	1.164729	0.038283

Supplementary Table S2C: Means, standard deviations and p-values of BMPR1A expression in the mesothelial cell lysate.

BMPR1A (Lys)	**CTR**	**D3**	**D5**	**D8**	**D11**
**average**	1	0.933162922	1.943695091	1.065478096	1.745238573
**standard deviation**	0	0.062514214	0.401511815	0.147172659	0.502379873

**Pairwise comparison**		**D1**	**D2**	**low limit**	**difference**	**up limit**	**p-values**
		1	2	-0.72955	0.066837	0.86322	0.998494
		1	3	-1.74008	-0.9437	-0.14731	0.019482
		1	4	-0.86186	-0.06548	0.730905	0.99861
		1	5	-1.54162	-0.74524	0.051144	0.069362
		2	3	-1.80692	-1.01053	-0.21415	0.012782
		2	4	-0.9287	-0.13232	0.664068	0.97989
		2	5	-1.60846	-0.81208	-0.01569	0.04521
		3	4	0.081834	0.878217	1.6746	0.02958
		3	5	-0.59793	0.198457	0.99484	0.918437
		4	5	-1.47614	-0.67976	0.116623	0.104985

Supplementary Table S2D: Means, standard deviations and p-values of BMPR2 expression in the mesothelial cell lysate.

BMPR2 (Lys)	**CTR**	**D3**	**D5**	**D8**	**D11**
**average**	1	1.458813986	1.768715273	1.776022401	2.43848052
**standard deviation**	0	0.138735767	0.332795637	0.76244494	0.523785246

**Pairwise comparison**		**D1**	**D2**	**low limit**	**difference**	**up limit**	**p-values**
		1	2	-1.65191	-0.45881	0.73428	0.716281
		1	3	-1.96181	-0.76872	0.424379	0.283313

		1	4	-1.96912	-0.77602	0.417072	0.275774
		1	5	-2.63157	-1.43848	-0.24539	0.01755
		2	3	-1.503	-0.3099	0.883193	0.906854
		2	4	-1.5103	-0.31721	0.875886	0.899743
		2	5	-2.17276	-0.97967	0.213428	0.123338
		3	4	-1.2004	-0.00731	1.185787	1
		3	5	-1.86286	-0.66977	0.523329	0.400665
		4	5	-1.85555	-0.66246	0.530636	0.410416

Supplementary Table S3

Supplementary Table S3A: Data for statistical analyzes of living and dead cells after BafA1 treatments.

	mean DensLiving	std DensDead	std DensLiving
CTR_{1B}	14.59069432	1.084789113	4.238286297
CTR_{2B}	17.81619409	0.89045129	7.801291132
$D3_{B}$	37.54703256	27.61669326	8.455465978
$D5_{B}$	16.32081455	25.24421275	14.49174426
$D8_{1B}$	16.84639751	30.48310862	6.594675039

Supplementary Table S3B: Code numbers for each group.

CTR_{1B}	1
CTR_{2B}	2
$D3_{B}$	3
$D5_{B}$	4
$D8_{1B}$	5

Supplementary Table S3C: Data for the statistical analysis on the number of dead cells.

D1	D2	low limit	difference	up limit	p-values
1	2	-15.693	1.833333	19.35966	0.998554
1	3	-45.0651	-26.6833	-8.30156	0.000716
1	4	-40.6651	-22.2833	-3.90156	0.008384
1	5	-48.3217	-30.4015	-12.4813	3.64E-05
2	3	-46.8984	-28.5167	-10.1349	0.000225
2	4	-42.4984	-24.1167	-5.7349	0.00318
2	5	-50.1551	-32.2348	-14.3146	9.16E-06
3	4	-14.7991	4.4	23.59913	0.971091
3	5	-22.4759	-3.71818	15.03953	0.98308
4	5	-26.8759	-8.11818	10.63953	0.762594

Supplementary Table S3D: Data for the statistical analysis on the number of living cells.

D1	D2	low limit	difference	up limit	p-values
1	2	-24.7576	-6.91667	10.92427	0.82822
1	3	-47.2451	-28.5333	-9.82161	0.000308
1	4	-19.9451	-1.23333	17.47839	0.999767
1	5	-22.8025	-4.56061	13.6813	0.960411
2	3	-40.3284	-21.6167	-2.90494	0.014065
2	4	-13.0284	5.683333	24.39506	0.921881
2	5	-15.8858	2.356061	20.59796	0.996705
3	4	7.756237	27.3	46.84376	0.001308
3	5	4.878307	23.97273	43.06715	0.005563
	5	-22.4217	-3.32727	15.76715	0.989583

Supplementary Table S4

Data for the statistical analyses about the signal intensity of JNK1-JNK2, Bcl-2, LC3B and active caspase-3.

Supplementary Table S4A: Means, standard deviations, code numbers and p-values of JNK1-JNK2 signal intensity.

Day	n	Mean	Std
CTR	21	1236.871	727.898
D3	34	1006.432	407.9073
D5	39	1143.798	365.3346
D8	32	1550.427	624.896
D11	27	1755.95	856.5664

**CTR**	**1**
**D3**	**2**
**D5**	**3**
**D8**	**4**
**D11**	**5**

Pairwise comparison		D1	D2	low limit	difference	up limit	p-values
		1	2	-19.015	14.53221	48.0794	0.761979
		1	3	-31.7598	0.956044	33.67185	0.999991
		1	4	-60.3633	-26.4182	7.526953	0.210206
		1	5	-67.5229	-32.3545	2.813933	0.088469
		2	3	-41.9366	-13.5762	14.78431	0.687627
		2	4	-70.7205	-40.9504	-11.1802	0.001644
		2	5	-78.0446	-46.8867	-15.7289	0.00039

		3	4	-56.2043	-27.3742	1.455877	0.072162
		3	5	-63.5714	-33.3105	-3.04965	0.022474
		4	5	-37.5222	-5.93634	25.64954	0.98614

Supplementary Table S4B: Means, standard deviations, code numbers and p-values of Bcl-2 signal intensity.

Day	n	Mean	Std
CTR	29	927.2835	235.681
D3	29	1194.689	517.0268
D5	35	1002.153	366.9538
D8	35	1362.379	421.054
D11	27	1644.243	793.116

**CTR**	**1**
**D3**	**2**
**D5**	**3**
**D8**	**4**
**D11**	**5**

**Pairwise comparison**		**D1**	**D2**	**low limit**	**difference**	**up limit**	**p-values**
		1	2	-51.018	-18.8621	13.2939	0.497286
		1	3	-35.5278	-4.78079	25.9662	0.993255
		1	4	-72.7849	-42.0379	-11.2909	0.001796
		1	5	-81.7543	-49.0083	-16.2623	0.000428
		2	3	-16.6657	14.08128	44.82827	0.722309
		2	4	-53.9229	-23.1759	7.571126	0.23944
		2	5	-62.8923	-30.1462	2.599805	0.088098
		3	4	-66.5274	-37.2571	-7.98688	0.004696
		3	5	-75.5911	-44.2275	-12.8639	0.001133
		4	5	-38.3339	-6.97037	24.3932	0.974162

Supplementary Table S4C: Data for the statistical analyses about the comparison of JNK1-JNK2 and Bcl-2 signal intensities.

CTR						
	'Source'	'SS'	'df'	'MS'	'Chi-sq'	'Prob>Chi-sq'
	'Groups'	271.4491	1	271.4491	1.277408	0.258382
	'Error'	10141.05	48	211.2719	[]	[]
	'Total'	10412.5	49	[]	[]	[]
D3						
	'Source'	'SS'	'df'	'MS'	'Chi-sq'	'Prob>Chi-sq'
	'Groups'	540.8032	1	540.8032	1.609533	0.204557
	'Error'	20291.2	61	332.6426	[]	[]

	'Total'	20832	62	[]	[]	[]
D5						
	'Source'	'SS'	'df'	'MS'	'Chi-sq'	'Prob>Chi-sq'
	'Groups'	1396.526	1	1396.526	3.019516	0.082268
	'Error'	32365.97	72	449.5274	[]	[]
	'Total'	33762.5	73	[]	[]	[]
D8						
	'Source'	'SS'	'df'	'MS'	'Chi-sq'	'Prob>Chi-sq'
	'Groups'	301.5598	1	301.5598	0.794275	0.372811
	'Error'	24756.44	65	380.8683	[]	[]
	'Total'	25058	66	[]	[]	[]
D11						
	'Source'	'SS'	'df'	'MS'	'Chi-sq'	'Prob>Chi-sq'
	'Groups'	73.5	1	73.5	0.29697	0.585788
	'Error'	13044	52	250.8462	[]	[]
	'Total'	13117.5	53	[]	[]	[]

Supplementary Table S4D: Means, standard deviations and statistical analysis of LC3B signal intensities.

Day	n	Mean	Std
$D5_B$	23	497.577	184.6813
$D8_{1B}$	26	220.0595	89.39902

'Source'	'SS'	'df'	'MS'	'Chi-sq'	'Prob>Chi-sq'
'Groups'	5039,625	1	5039,625	24,68639862	6,75E-07
'Error'	4759,375	47	101,2633	[]	[]
'Total'	9799	48	[]	[]	[]

Supplementary Table S4E: Means, standard deviations, code numbers and statistical analysis of active caspase-3 signal intensities.

Day	n	Mean	SD
CTR	24	283.5031	128.0244
D8	28	352.5409	148.697
$D8_{2B}$	30	321.8593	126.4881

**CTR**	**1**
**D8**	**2**
**$D8_{2B}$**	**3**

Pairwise comparison		D1	D2	low limit	difference	up limit	p-values
		1	2	-26.5323	-11.006	4.520412	0.220218
		1	3	-22.2106	-6.925	8.360631	0.537887
		2	3	-10.5856	4.080952	18.74747	0.791157
